# Enhancing Emergency Medicine Resident Education: A Weekly Education Series to Augment Electrocardiogram Education

**DOI:** 10.5070/M5.52141

**Published:** 2026-01-31

**Authors:** Tyler West, Jarren Adam, Kevin Watkins

**Affiliations:** * Cleveland Clinic Akron General, Department of Emergency Medicine, Akron, OH

## Abstract

**Audience and Type of Curriculum:**

This electrocardiogram (ECG) curriculum was designed for residents of all levels.

**Length of Curriculum:**

The curriculum runs over 1.5 years and is repeated, so that there will be repetition during a resident’s training.

**Introduction:**

Electrocardiogram interpretation is a vital skill for emergency physicians. Formal ECG education in emergency medicine (EM) often consists of a few conference lectures, with the majority of education relegated to the clinical environment. However, this often leaves significant gaps in education, as a full curriculum cannot be implemented within limited conference time.

**Educational Goals:**

The goals of the curriculum were to establish an asynchronous ECG curriculum to help improve standardization of EM resident education, expose EM residents to a more comprehensive ECG curriculum, increase active learning in this arena, and decrease the administrative burden while not dedicating further conference time towards ECG education.

**Educational Methods:**

The educational strategies used in this curriculum include weekly case emails with instructional content located on a Google Site. These were added to conference lectures that were standard ECG education prior to this initiative. The first year of the augmented curriculum added only the weekly ECG emails with a follow-up answer email, and the website was created for the second year of curriculum implementation.

**Research Methods:**

The educational content was assessed by the learners via a survey to gauge resident satisfaction as well as level of engagement and barriers to use. Additionally, the content was assessed via resident testing at the end of their second year so that they had completed a complete cycle of the curriculum.

**Results:**

The asynchronous curriculum improved resident test scores with the addition of the weekly emails from an average of 70% and pass rate of 58% to 82% and 92%, respectively (p=0.012). The addition of the informational website did not further improve scores, though it decreased variation in scores. The residents found the curriculum useful for their education.

**Discussion:**

Curriculum implementation was successful to improve on resident ECG education, both in terms of objective testing as well as resident feedback. It is a sustainable curriculum with methodology that requires little faculty time after setup; the maintenance required for the curriculum mostly consists of updating resident contacts as classes graduate. However, the setup time was significant; despite this, the authors believe this is a time-effective method of educational programming given the little ongoing time requirements.

**Topics:**

Electrocardiogram, curriculum development.

## USER GUIDE

**Table:** 

List of Resources:	
Abstract	1
User Guide	3
Didactics and Hands on Curriculum Chart	6
Appendix A: Overview of logistics for system automatization	9
Appendix B: Google Sheets and Forms	10
Appendix C: Sample topic page from the website	12
Appendix D: Weekly EKG Cases and Answers	13

**Learner Audience:**

Interns, Junior Residents, Senior Residents

**Length of Curriculum:**

The longitudinal curriculum spans a 1.5-year period and is recurring.

**Topics:**

Electrocardiogram, curriculum development.

**Objectives:**

By the end of this curriculum, learners will be able to:

1. Interpret normal ECGs as well as basic ECG pathology
2. Interpret more advanced ECGs found with emergent medical conditions
3. Apply their interpretation in the setting of triage common to emergency departments

### Brief introduction

The ability to interpret electrocardiograms in real time is a vital skill in emergency medicine for patient care, aiding in the identification of acute myocardial injury, dysrhythmias, and toxidromes.^1^,^2^ Traditionally, ECG education has relied on clinical training, commonly with the addition of several conference lectures. Increasingly, medical schools and residencies are incorporating formal ECG training utilizing web-based, face-to-face curriculum, and clinical scenarios.^1^,^3^–^9^ Some studies suggest that web-based designs are not inferior to lecture-based teaching and may even improve interpretation. 1,2,3,7,10–15 Combining online-based methods with traditional ones has shown improvement in retention and interpretation of ECGs for medical students and residents.^2^ This also allows spacing of instruction over time, which was previously found to be beneficial.^16^ Other studies have shown that the format of teaching may be less important than the type of assessment, with summative assessments improving interpretation over formatives ones.^7^

### Problem identification, general and targeted needs assessment

Despite its importance, many EM residents start their training at different levels of comfort with interpretation and report feeling their ECG training is inadequate.^17^ Additionally, there is no definitive list of diagnoses or method to obtain competency in interpretation, despite the fact that model of clinical practice of emergency medicine lists ECG interpretation as a skill integral to the practice.^2^,^18^–^20^ While some publications have created lists of diagnoses, it is unlikely that a trainee would encounter the full list during training, necessitating supplemental education.^2^ Furthermore, protected conference time is limited to administer a comprehensive ECG education necessary for clinical practice, leading programs to rely on bedside teaching that may not cover all useful topics.

With the plethora of information available to EM residents in the format of emails, podcasts, online modules, social media, and blogs an online format for ECG training may enhance residents’ interpretation abilities and, consequently, patient care.^13^,^21^,^22^,^23^ Despite the increasing use of online resources in emergency medicine residencies, there is limited comprehensive, cohesive curriculum designed for web-based teaching.^21^ While Foundations of Emergency Medicine initiated an online curriculum available for residency programs, this study aimed to enhance it by adding additional topics beneficial for an emergency medicine career.^17^ This study assesses the competency of ECG interpretation by adding an expanded, online weekly curriculum as an adjunct to traditional lecture-based format already being used in a three-year emergency medicine residency. Additionally, the setup allows for efficient use of faculty time.

### Goals of the curriculum

The goals of the curriculum were to establish an asynchronous ECG curriculum to help improve standardization of EM resident education within the residency program, expose EM residents to a more comprehensive ECG curriculum that expanded from that of Foundations of Emergency Medicine, increase active learning in this arena, and decrease the administrative burden of a continual, asynchronous curriculum while not dedicating further conference time towards ECG education given the limited time available relative to the breadth of information.

### Objectives of the curriculum

By the end of this curriculum, learners will be able to:

1. Interpret normal ECGs as well as basic ECG pathology
2. Interpret more advanced ECGs found with emergent medical conditions
3. Apply their interpretation in the setting of triage common to emergency departments

### Educational Strategies

See curriculum chart

### Results and tips for successful implementation

The curriculum was first developed in 2020 after the first cohort of residents took the ECG test. The addition of the website as a base of continual study for the residents was introduced in the subsequent year. The curriculum was tested both monitoring the results of an ECG test that was administered to PGY-2 residents over a period of 4 years as the curriculum was initially introduced and then expansion with the website. In total, 34 residents took the ECG test over this timeframe as there are typically 11 residents in each year of the program with the exception that an additional resident was present in the first cohort before implementation of the curriculum. The PGY-2 year was chosen as they would have completed a full cycle of the curriculum and begin to sign ECGs clinically in the department at the end of the training year. The tests showed improved interpretation skills after implementation of the curriculum with the emails with mean scores improving from 70% to 82% and pass rate improving from 58% to 92% (p=0.012), though did not show statistical significance for further improvement after implementation of the website. However, the variance of test results did decrease significantly after implementation of the website, going from 12% to 4% in the first year. Additionally, two voluntary surveys were sent to residents of all classes, as residents in all years of training participate in this education, to gauge resident satisfaction as well as level of engagement and barriers to use. The first survey was sent prior to initiation of the website and had 20 respondents, the majority (17) of whom reported noting benefit from the email curriculum. However, many actively participated in few of the email responses. The second survey was sent out after implementation of the website to further assess it, in particular. The second survey also had 20 respondents that showed many did use the website, though again many had limited active participation in the email responses. Commonly reported reasons for lack of participation included laziness, emails lost amongst others, and not being comfortable sharing their interpretation despite the anonymous nature of the response recordings.

We have continued to run the program in subsequent years. One faculty member created the curriculum and its implementation. The initial setup requires significant administrative time that primarily involves creation of the website and its content; however, the maintenance required after initial setup is primarily changing resident emails every year, monitoring their answer submissions to guide potential changes, and administering the ECG test as the emails are automatically sent every week using this system as long it is updated after every cycle.

### Evaluation and Feedback

The only modifications made in subsequent years were removing restricted access to the website with password submission, changing some of the ECG images that were felt to be improvements compared to priors, and reinforcing the fact that responses are anonymous.

### Associated Content

Appendix A: Overview of Logistics for System Automatization

Appendix B: Google Sheets and Forms

Appendix C: Sample Topic Page from the Website

Appendix D: Weekly EKG Cases and Answers

## Supplementary Information





## Figures and Tables

**Table:** 

Topic	Recommended Educational Strategy	Educational Content	Objectives	Learners	Timing, Resources Needed	Recommended Assessment, Milestones Addressed
Basics	Weekly email with ECG and brief case Website with topic pages to provide answers	Atrial Enlargement (Week 5) Left Ventricular Hypertrophy (Week 8) Right Ventricular Hypertrophy (Week 9) Axis Deviation (Week 6) QT Interval (Week 7) Lead Misplacement (Week 10) Dextrocardia (Week 11) Sinus Arrhythmia (Week 12) Pediatric EKGs (Week 13) Junctional Rhythms (Week 27) Ventricular Escape (Week 28) Premature Contractions (Week 29) Atrial Septal Defects (Week 30) Low Voltage/Electrical Alternans (Week 58)	The learner will understand basic normal EKGs as well as pathology.	All PGYs	1 minute (email) Instructors: 1 Equipment: Google Drive, internet	Milestone: PC3 Assessment: EKG Quiz
Blocks	Weekly email with ECG and brief case Website with topic pages to provide answers	1* AV Block (Week 1) Mobitz I Block (Week 3) Mobitz II Block (Week 4) 3* AV Block (Week 2) Right Bundle Branch Block (Week 23) Left Bundle Branch Block (Week 24) Fascicular Blocks (Week 25) Nonspecific Intraventricular Conduction Delay (Week 26)	The learner will understand the variety of AV and His-Purkinje blocks.	All PGYs	1 minute (email) Instructors: 1 Equipment: Google Drive, internet	Milestone: PC3 Assessment: EKG Quiz
Narrow Complex Tachycardias	Weekly email with ECG and brief case Website with topic pages to provide answers	Atrial Fibrillation (Week 52) Ashman Phenomenon (Week 53) Atrial Flutter (Week 50) Ectopic Atrial Rhythm (Week 49) Multifocal Atrial Tachycardia (Week 51) AV Nodal Reentrant Tachycardia (Week 48) Tachybrady syndrome (Week 54)	The learner will understand the different narrow complex tachycardias and how to differentiate them.	All PGYs	1 minute (email) Instructors: 1 Equipment: Google Drive, internet	Milestone: PC3 Assessment: EKG Quiz
Wide Complex Tachycardias	Weekly email with ECG and brief case Website with topic pages to provide answers	Ventricular Fibrillation (Week 67) Ventricular Tachycardia Basics (Week 68) First 3 Brugada Criteria (Week 69) Brugada Morphologic Criteria (Week 70) Vereckei Algorithm (Week 72) R Wave Peak Time (Week 71) Griffith Algorithm (Week 73) Idiopathic Ventricular Tachycardia (Week 74)	The learner will understand wide complex tachycardias and how to differentiate VT from SVT with aberrancy.	All PGYs	1 minute (email) Instructors: 1 Equipment: Google rive, internet	Milestone: PC3 Assessment: EKG Quiz
STEMI	Weekly email with ECG and brief case Website with topic pages to provide answers	STEMI Basics (Week 14) Anterolateral STEMI (Week 15) Wellens’ Syndrome (Week 16) DeWinter’s T Waves (Week 18) Inferior STEMI (Week 20) Posterior STEMI (Week 21) Left Main Coronary Artery Occlusion (Week 19) Hyperacute T Waves (Week 17) Sharkfin STEMI (Week 22)	The learner will understand the basics of STEMI patterns and the different types.	All PGYs	1 minute (email) Instructors: 1 Equipment: Google Drive, internet	Milestone: PC3 Assessment: EKG Quiz
Other Ischemia	Weekly email with ECG and brief case Website with topic pages to provide answers	T Wave Inversions (Week 32) ST Depressions (Week 31) Poor R Wave Progression (Week 33) Pathologic Q Waves (Week 34) QRS Fragmentation (Week 35) Terminal QRS Distortion (Week 36)	The learner will understand EKG changes consistent with ischemia besides that of ST elevation.	All PGYs	1 minute (email) Instructors: 1 Equipment: Google Drive, internet	Milestone: PC3 Assessment: EKG Quiz
Ischemia Mimics	Weekly email with ECG and brief case Website with topic pages to provide answers	Ventricular Aneurysm (Week 60) Pericarditis (Week 61) Early Repolarization (Week 62) Benign T Wave Inversion (Week 59) Intracranial Hypertension (Week 55) Atrial Repolarization (Week 57) Pulmonary Embolism (Week 56)	The learner will understand the different mimics of acute coronary syndrome.	All PGYs	1 minute (email) Instructors: 1 Equipment: Google Drive, internet	Milestone: PC3 Assessment: EKG Quiz
Tox/Metabolic	Weekly email with ECG and brief case Website with topic pages to provide answers	Hypocalcemia (Week 37) Hypercalcemia (Week 38) Hypokalemia/Hypomagnesemia (Week 39) Hyperkalemia (Week 40) Digoxin (Week 42) Sodium Channel Blocker Toxicity (Week 43) Hypothermia (Week 41)	The learner will understand the variety of EKG changes seen in toxicologic and metabolic emergencies.	All PGYs	1 minute (email) Instructors: 1 Equipment: Google Drive, internet	Milestone: PC3 Assessment: EKG Quiz
Syncope	Weekly email with ECG and brief case Website with topic pages to provide answers	Hypertrophic ardiomyopathy (Week 44) Arrhythmogenic Right Ventricular Cardiomyopathy (Week 46) Brugada Syndrome (Week 45) Wolff-Parkinson White (Week 47)	The learner will understand the EKG findings of syncopal syndromes.	All PGYs	1 minute (email) Instructors: 1 Equipment: Google Drive, internet	Milestone: PC3 Assessment: EKG Quiz
Pacemaker	Weekly email with ECG and brief case Website with topic pages to provide answers	Pacemaker Basics (Week 63) Failure to Sense (Week 65) Failure to Pace (Week 64) Pacemaker Tachydysrhythmias (Week 66)	The learner will understand the normal and abnormal findings seen in pacemaker EKGs.	All PGYs	1 minute (email) Instructors: 1 Equipment: Google Drive, internet	Milestone: PC3 Assessment: EKG Quiz

**Table:** 

Week 1:	1* AV Block
Week 2:	3* AV Block
Week 3:	Mobitz I Block
Week 4:	Mobitz II Block
Week 5:	Atrial Enlargement
Week 6:	Axis Deviation
Week 7:	QT Interval
Week 8:	Left Ventricular Hypertrophy
Week 9:	Right Ventricular Hypertrophy
Week 10:	Lead Misplacement
Week 11:	Dextrocardia
Week 12:	Sinus Arrhythmia
Week 13:	Pediatric EKGs
Week 14:	STEMI Basics
Week 15:	Anterolateral STEMI
Week 16:	Wellens’ Syndrome
Week 17:	Hyperacute T Waves
Week 18:	DeWinter’s T Waves
Week 19:	Left Main Coronary Artery Occlusion
Week 20:	Inferior STEMI
Week 21:	Posterior STEMI
Week 22:	Sharkfin STEMI
Week 23:	Right Bundle Branch Block
Week 24:	Left Bundle Branch Block
Week 25:	Fascicular Blocks
Week 26:	Nonspecific Intraventricular Conduction Delay
Week 27:	Junctional Rhythms
Week 28:	Ventricular Escape
Week 29:	Premature Contractions
Week 30:	Atrial Septal Defects
Week 31:	ST Depressions
Week 32:	T Wave Inversions
Week 33:	Poor R Wave Progression
Week 34:	Pathologic Q Waves
Week 35:	QRS Fragmentation
Week 36:	Terminal QRS Distortion
Week 37:	Hypocalcemia
Week 38:	Hypercalcemia
Week 39:	Hypokalemia/Hypomagnesemia
Week 40:	Hyperkalemia
Week 41:	Hypothermia
Week 42:	Digoxin
Week 43:	Sodium Channel Blocker Toxicity
Week 44:	Hypertrophic Cardiomyopathy
Week 45:	Brugada Syndrome
Week 46:	Arrhythmogenic Right Ventricular Cardiomyopathy
Week 47:	Wolff-Parkinson White
Week 48:	AV Nodal Reentrant Tachycardia
Week 49:	Ectopic Atrial Rhythm
Week 50:	Atrial Flutter
Week 51:	Multifocal Atrial Tachycardia
Week 52:	Atrial Fibrillation
Week 53:	Ashman Phenomenon
Week 54:	Tachybrady syndrome
Week 55:	Intracranial Hypertension
Week 56:	Pulmonary Embolism
Week 57:	Atrial Repolarization
Week 58:	Low Voltage/Electrical Alternans
Week 59:	Benign T Wave Inversion
Week 60:	Ventricular Aneurysm
Week 61:	Pericarditis
Week 62:	Early Repolarization
Week 63:	Pacemaker Basics
Week 64:	Failure to Pace
Week 65:	Failure to Sense
Week 66:	Pacemaker Tachydysrhythmias
Week 67:	Ventricular Fibrillation
Week 68:	Ventricular Tachycardia Basics
Week 69:	First 3 Brugada Criteria
Week 70:	Brugada Morphologic Criteria
Week 71:	R Wave Peak Time
Week 72:	Vereckei Algorithm
Week 73:	Griffith Algorithm
Week 74:	Idiopathic Ventricular Tachycardia

**Figure:**
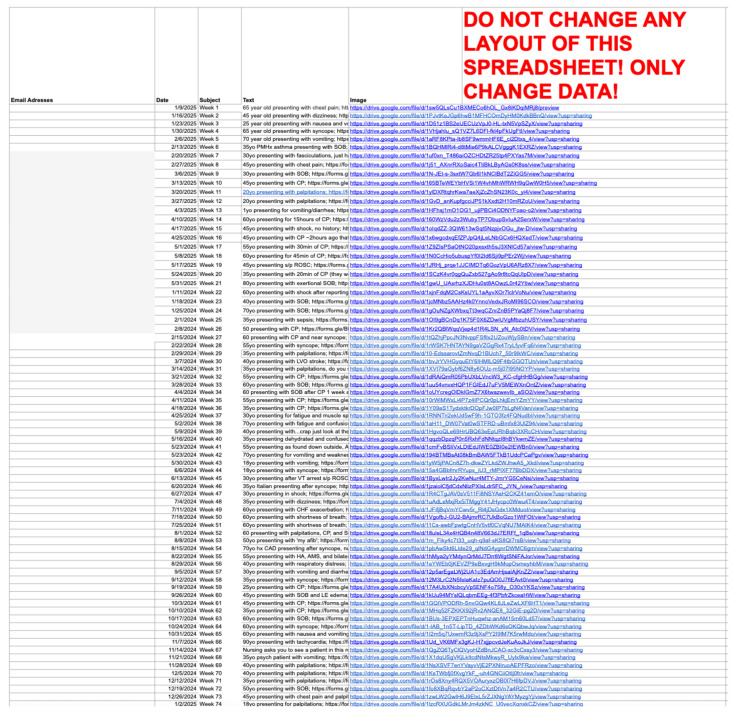
Google Form for resident answers, programs can make a copy and responder link to compile their resident’s answers.

**Figure:**
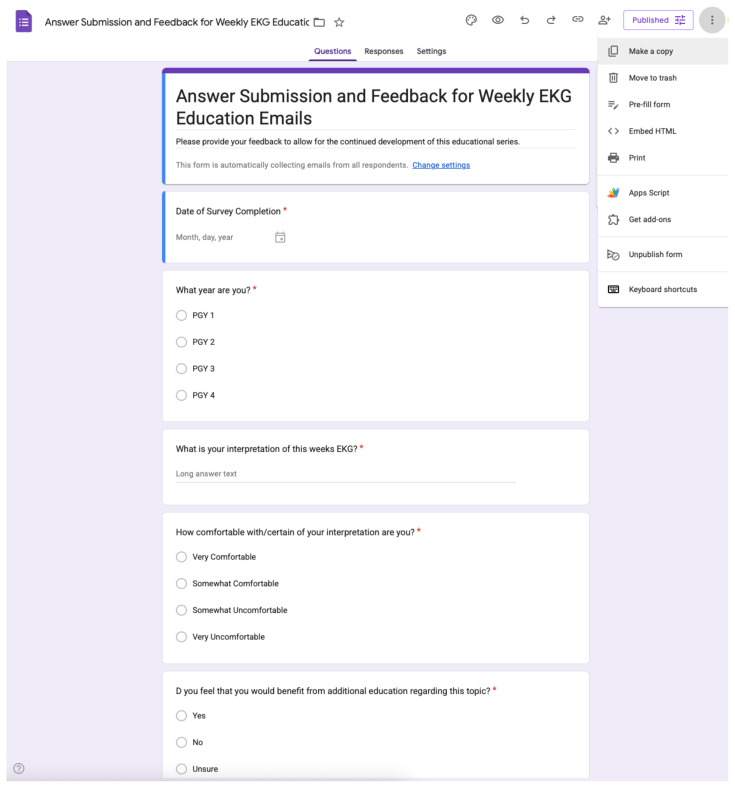
https://docs.google.com/forms/d/1ucdRoPcGMFfjDy1f6MnyFADJP3W4kjiZs5Rh6UOkI_c/edit

**Figure:**
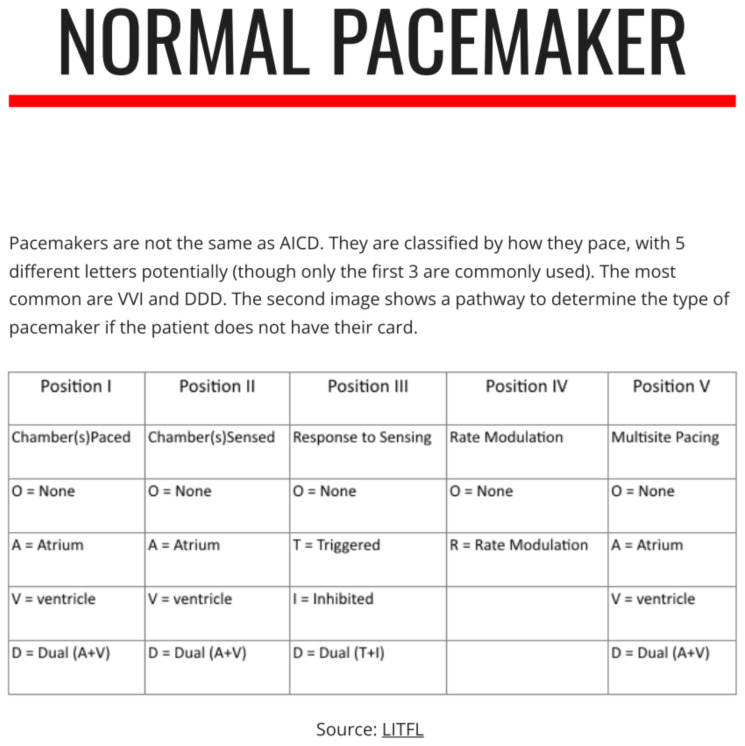
https://www.ccagem.com/ekg-epistemology

**Figure:**
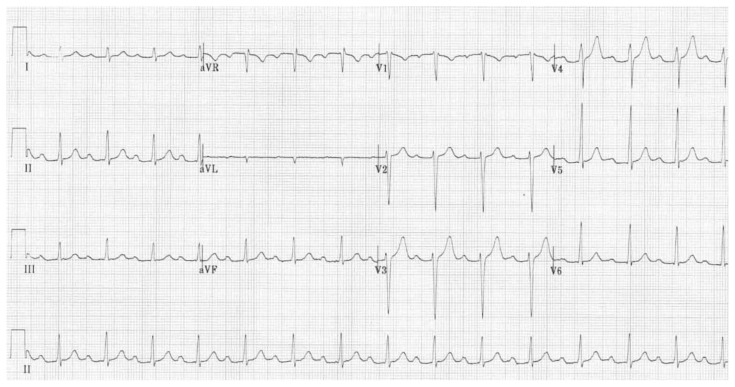
Larkin J, Buttner R. Example 3, First Degree Atrioventricular (AV) Block. In: Life in the Fast Lane. CC BY-NC-SA. https://litfl.com/first-degree-heart-block-ecg-library/

**Figure:**
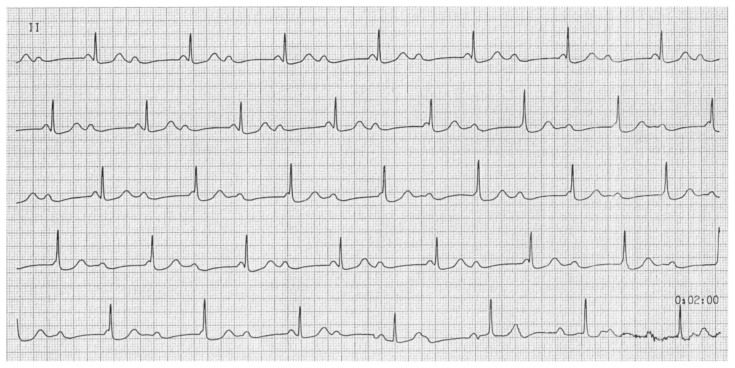
Larkin J, Buttner R. Example 4, Complete Heart Block with Isorhythmic AV Dissociation (long rhythm strip). In: Life in the Fast Lane. CC BY-NC-SA. https://litfl.com/av-block-3rd-degree-complete-heart-block/

**Figure:**
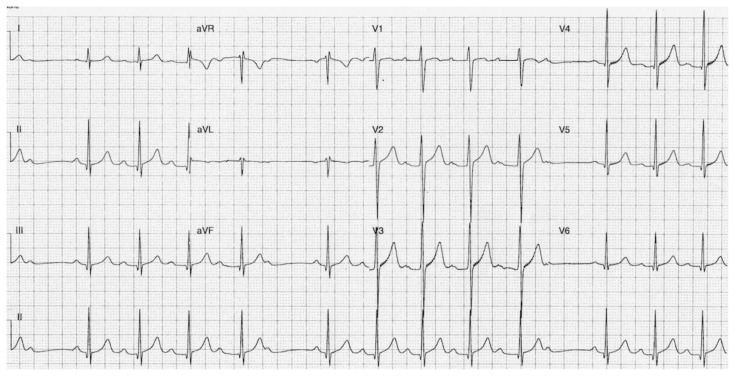
Burns E, Buttner R. Example 2, Mobitz I AV block. In: Life in the Fast Lane. CC BY-NC-SA. https://litfl.com/av-block-2nd-degree-mobitz-i-wenckebach-phenomenon/

**Figure:**
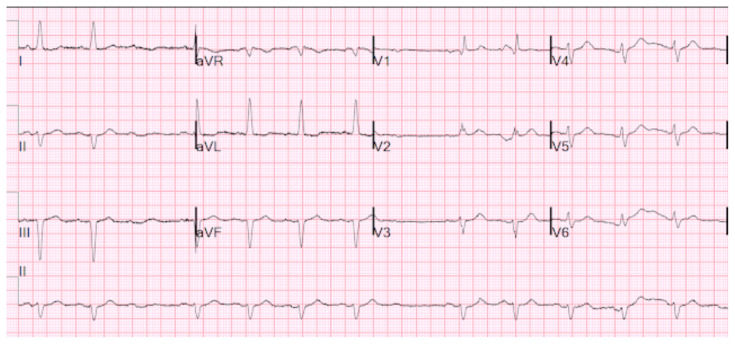
Smith S. A Pre-operative ECG on a patient with a hip fracture. In: Dr. Smith’s ECG Blog. CC BY-NC. https://drsmithsecgblog.com/a-pre-operative-ecg-on-patient-with-hip/

**Figure:**
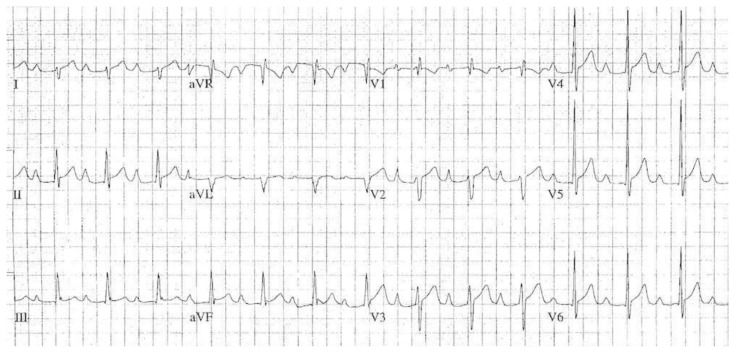
Burns E, Buttner R. Example 3, Biatrial enlargement. In: Life in the Fast Lane. CC BY-NC-SA. https://litfl.com/biatrial-enlargement-ecg-library/

**Figure:**
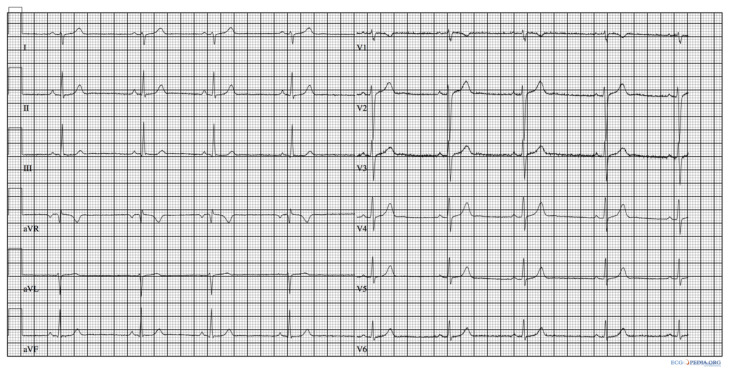
van der Bilt, I. QRS Axis. In: ECGPedia. CC BY-NC-SA. https://en.ecgpedia.org/wiki/QRS_axis#Right_axis_deviation

**Figure:**
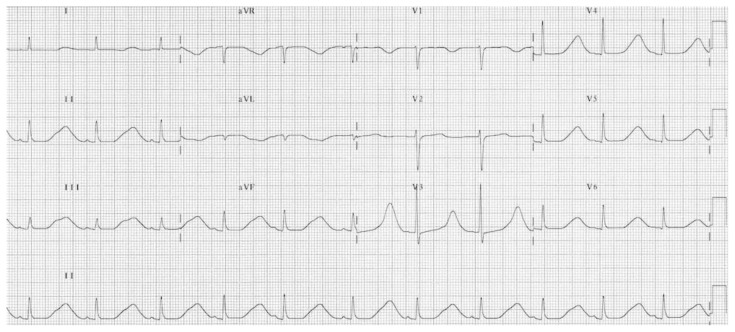
Burns E, Buttner R. Hypocalcaemia. In: Life in the Fast Lane. CC BY-NC-SA. https://litfl.com/qt-interval-ecg-library/

**Figure:**
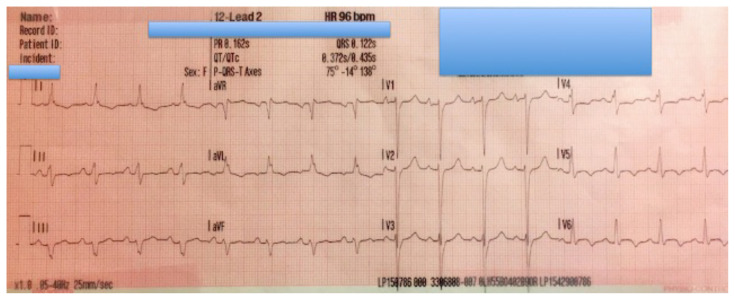
Smith S. Occlusion Myocardial Infarction (OMI). Can be Diagnosed by “Pseudonormalization of ST Segments.” In: Dr. Smith’s ECG Blog. CC BY-NC. https://drsmithsecgblog.com/omi-can-be-diagnosed-by

**Figure:**
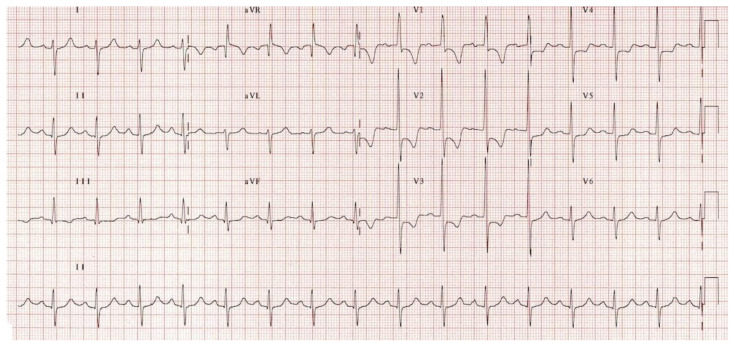
Burns E, Buttner R. Example 1, Right Ventricular Hypertrophy. In: Life in the Fast Lane. CC BY-NC-SA. https://litfl.com/right-ventricular-hypertrophy-rvh-ecg-library/

**Figure:**
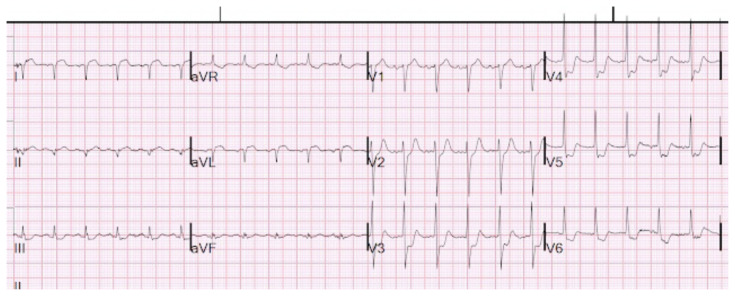
Smith S. ST Elevation in aVL with reciprocal ST Depression in inferior leads and Diffuse ST depression. In: Dr. Smith’s ECG Blog. CC BY-NC. https://drsmithsecgblog.com/st-elevation-in-avl-with-reciprocal-st/

**Figure:**
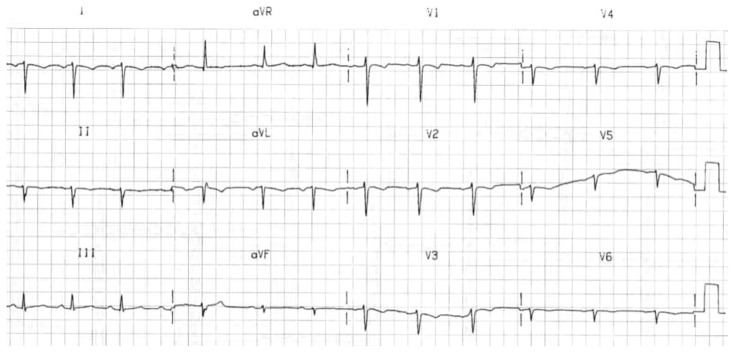
Nickson C, Buttner R. Example 1, Dextrocardia. In: Life in the Fast Lane. CC BY-NC-SA. https://litfl.com/dextrocardia-ecg-library/

**Figure:**
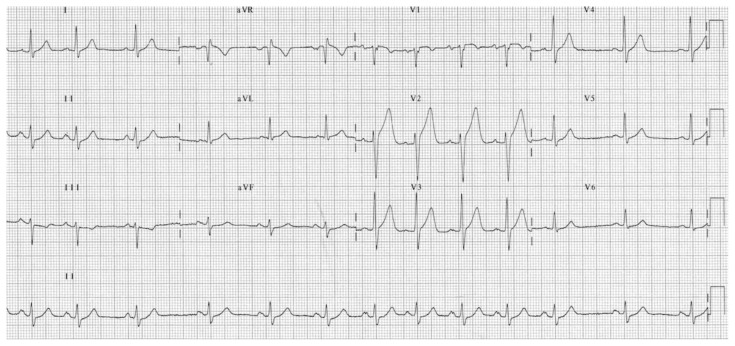
Burns E. Sinus Arrhythmia. In: Life in the Fast Lane. CC BY-NC-SA. https://litfl.com/sinus-arrhythmia-ecg-library/

**Figure:**
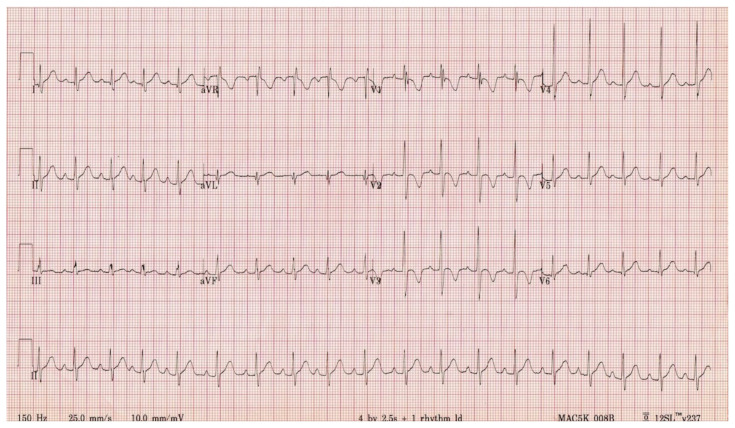
Burns E, Buttner R. Example 1, Normal paediatric ECG. In: Life in the Fast Lane. CC BY-NC-SA. https://litfl.com/normal-paediatric-ecg

**Figure:**
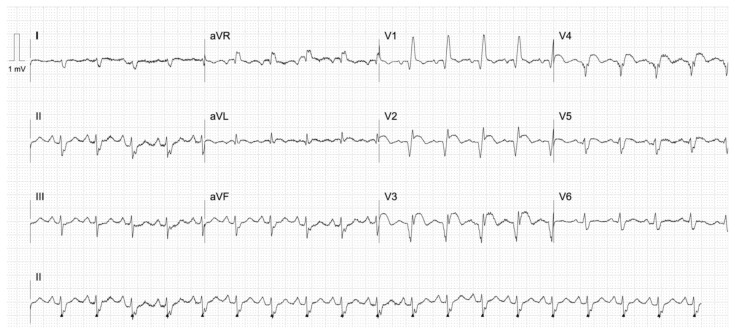
Burns E, Buttner R. Left Ventricular Aneurysm. In: Life in the Fast Lane. CC BY-NC-SA. https://litfl.com/left-ventricular-aneursym-ecg-library/

**Figure:**
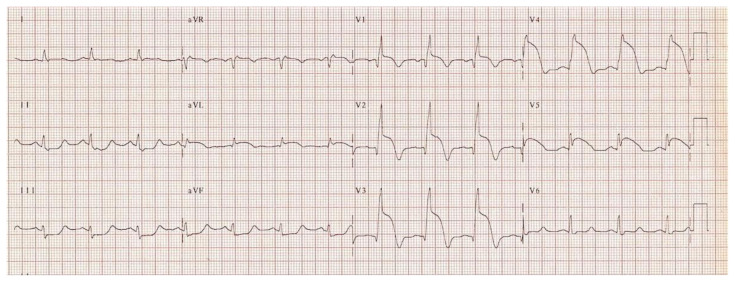
Burns E, Buttner R. Example 9, Anterior Myocardial Infarction. In: Life in the Fast Lane. CC BY-NC-SA. https://litfl.com/anterior-myocardial-infarction-ecg-library/

**Figure:**
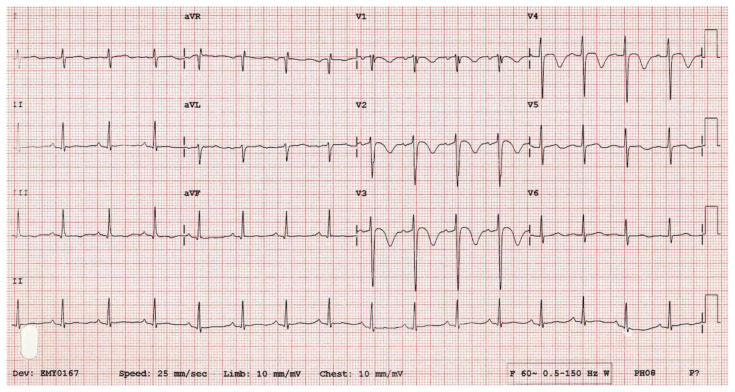
Cadogan M, Buttner R. Wellens Syndrome. In: Life in the Fast Lane. CC BY-NC-SA. https://litfl.com/wellens-syndrome-ecg-library

**Figure:**
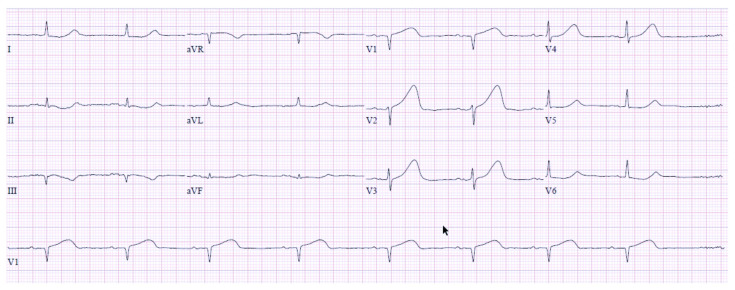
Santistevan J. ECG Pointers: Hyperacute T-Waves. In: emDocs. CC BY-NC-SA. https://www.emdocs.net/ecg-pointers-hyperacute-t-waves/

**Figure:**
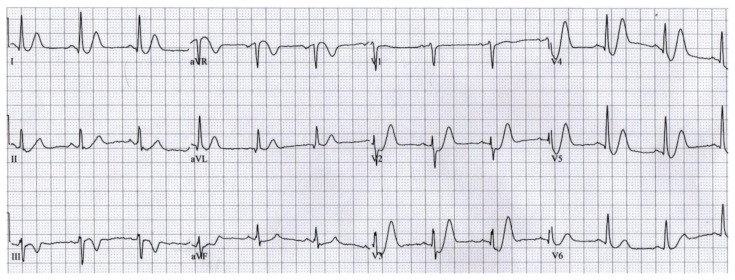
Cadogan M, Buttner R. Example 2, De Winter T waves. In: Life in the Fast Lane. CC BY-NC-SA. https://litfl.com/de-winter-t-wave/

**Figure:**
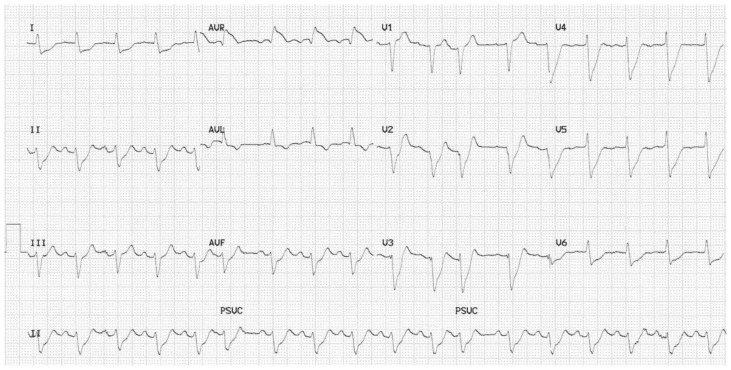
Burns E, Buttner R. Example 10, left main coronary artery (LMCA) sub-total occlusion. In: Life in the Fast Lane. CC BY-NC-SA. https://litfl.com/st-elevation-in-avr/

**Figure:**
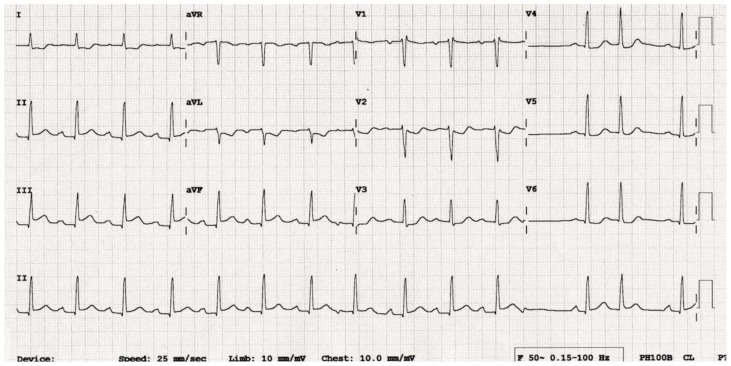
Burns E, Buttner R. Example 4a, Right Ventricular Infarction. In: Life in the Fast Lane. CC BY-NC-SA. https://litfl.com/right-ventricular-infarction-ecg-library/

**Figure:**
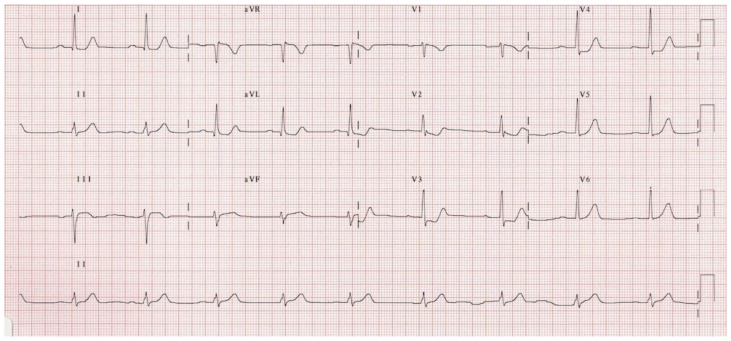
Buttner R, Burns E. Example 2a, Posterior Myocardial Infarction. In: Life in the Fast Lane. CC BY-NC-SA. https://litfl.com/posterior-myocardial-infarction-ecg-library/

**Figure:**
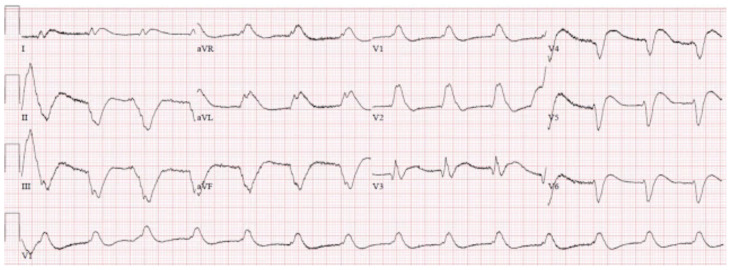
Myers P. Shark Fin morphology recognized only by EM physician. In: Dr. Smith’s ECG Blog. CC BY-NC. https://drsmithsecgblog.com/shark-fin-morphology-recognized-only-by/

**Figure:**
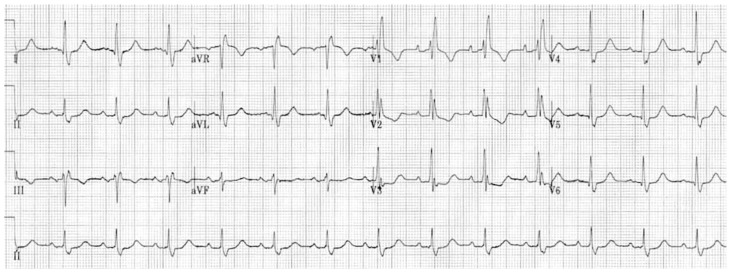
Burns E, Buttner R. Example 2, Right Bundle Branch Block (RBBB). In: Life in the Fast Lane. CC BY-NC-SA. https://litfl.com/right-bundle-branch-block-rbbb-ecg-library/

**Figure:**
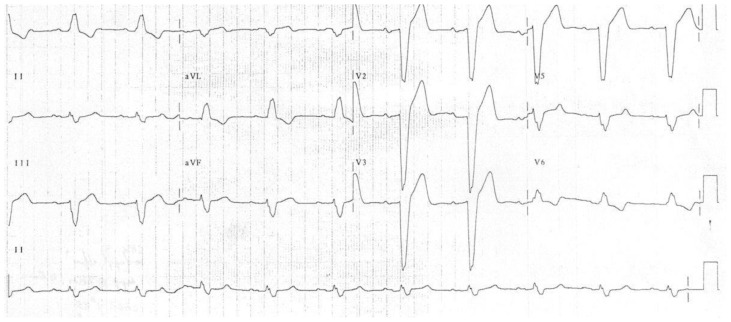
Burns E, Buttner R. Example 3, Left Bundle Branch Block. In: Life in the Fast Lane. CC BY-NC-SA. https://litfl.com/left-bundle-branch-block-lbbb-ecg-library/

**Figure:**
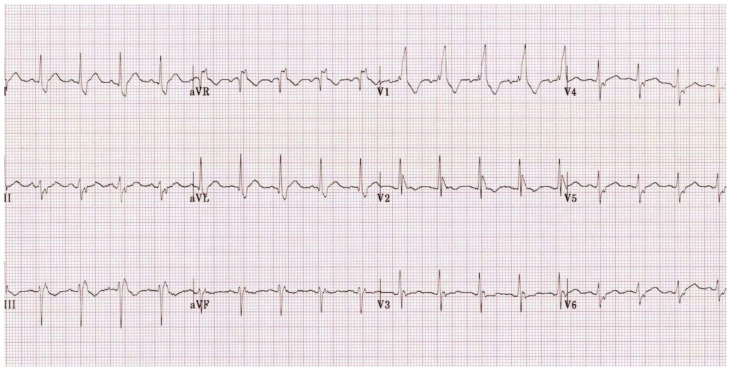
Burns E, Buttner R. Typical bifascicular block pattern. In: Life in the Fast Lane. CC BY-NC-SA. https://litfl.com/bifascicular-block-ecg-library

**Figure:**
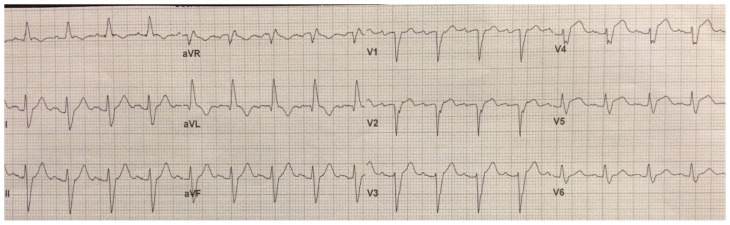
Smith S. Prolonged chest pain and Intraventricular Conduction Delay similar to Left Bundle Branch Block. In: Dr. Smith’s ECG Blog. CC BY-NC. https://drsmithsecgblog.com/prolonged-chest-pain-and/

**Figure:**
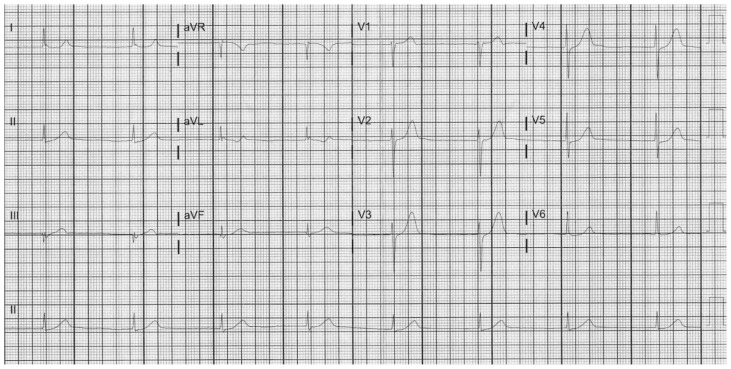
Smith S. Junctional Bradycardia. In: Dr. Smith’s ECG Blog. CC BY-NC. https://drsmithsecgblog.com/middle-aged-male-with-burning-chest/

**Figure:**
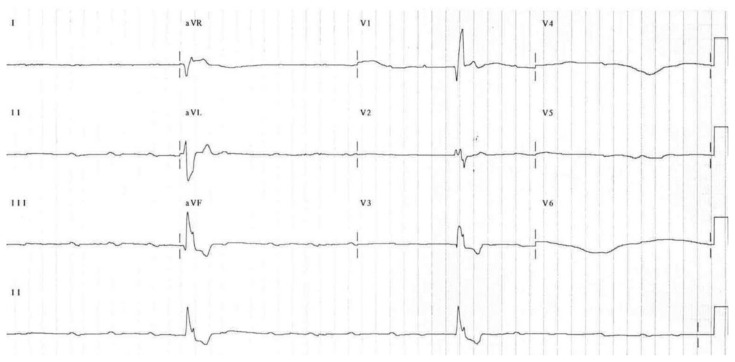
Burns E, Buttner R. Complete heart block with a ventricular escape rhythm. In: Life in the Fast Lane. CC BY-NC-SA. https://litfl.com/ventricular-escape-rhythm-ecg-library/

**Figure:**
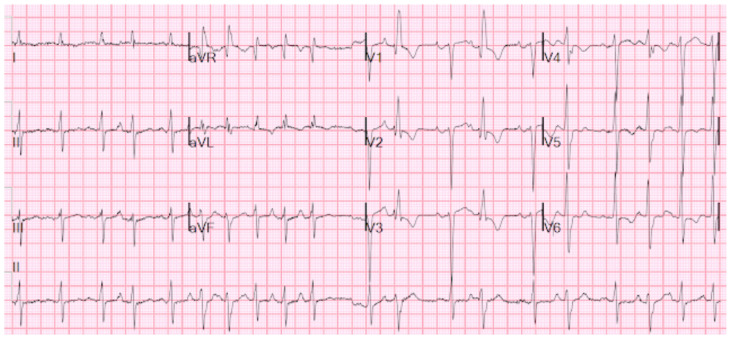
Smith S. Atrial fibrillation Multifocal Atrial Tachycardia? Don’t look at computer read until AFTER you interpret! CC BY-NC. In: Dr. Smith’s ECG Blog. https://drsmithsecgblog.com/atrial-fibrillation-multifocal-atrial/

**Figure:**
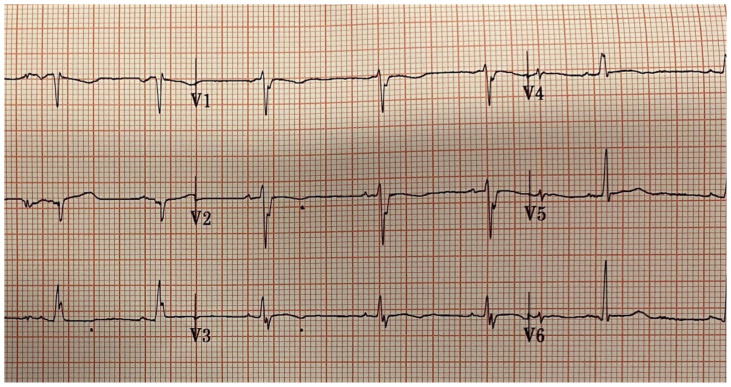
Smith S. A young woman with epigastric pain. ECG Crochetage sign? What is the significance? In: Dr. Smith’s ECG Blog. CC BY-NC. https://drsmithsecgblog.com/a-young-woman-with-epigastric-pain-ecg/

**Figure:**
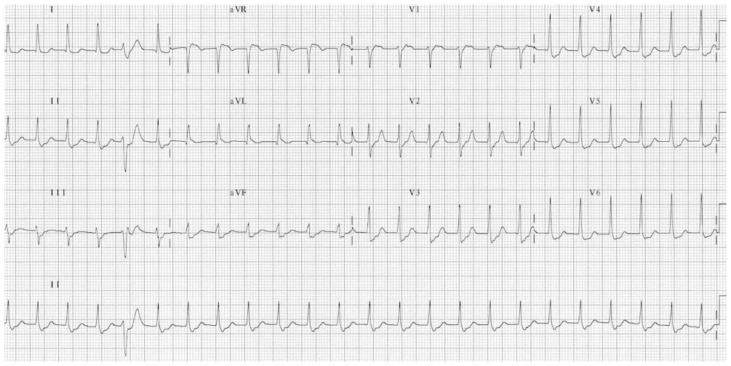
Burns E, Buttner R. Supraventricular tachycardia. In: Life in the Fast Lane. CC BY-NC-SA. https://litfl.com/st-segment-ecg-library

**Figure:**
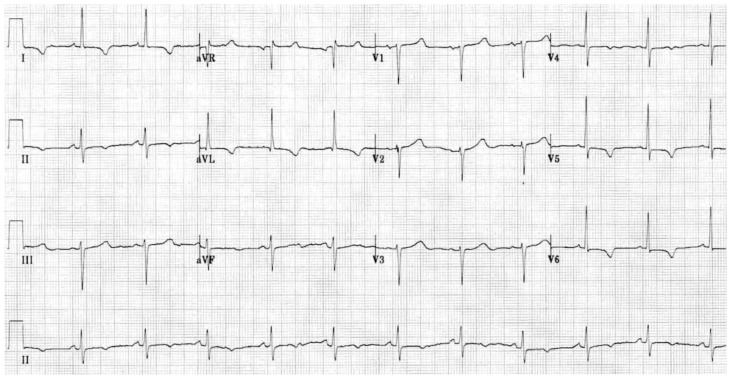
Burns E, Cadogan M. T Wave Inversion. CC BY-NC-SA. In: Life in the Fast Lane. https://litfl.com/myocardial-ischaemia-ecg-library/

**Figure:**
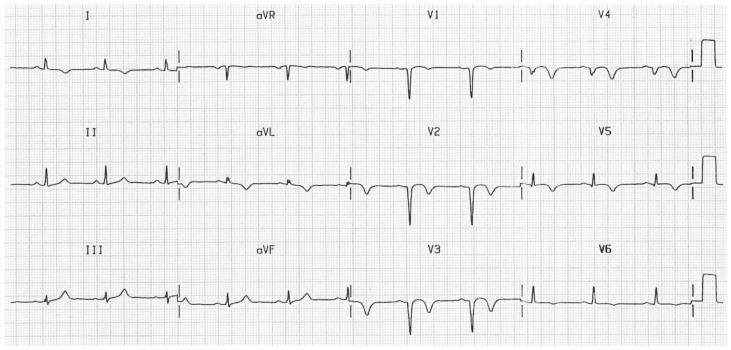
Burns E, Buttner R. Poor R wave progression secondary to prior anteroseptal myocardial infarction (MI). In: Life in the Fast Lane. CC BY-NC-SA. https://litfl.com/poor-r-wave-progression-prwp-ecg-library/

**Figure:**
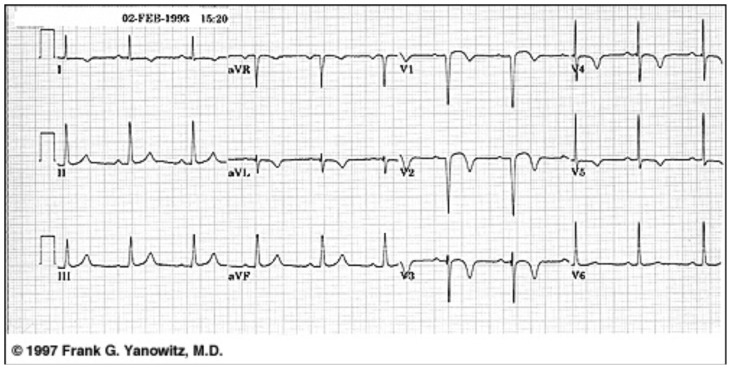
Yanowitz F. Anteroseptal MI. CC BY-NC-ND. In: ECG Learning Center. https://ecg.utah.edu/lesson/9#Ant_MI

**Figure:**
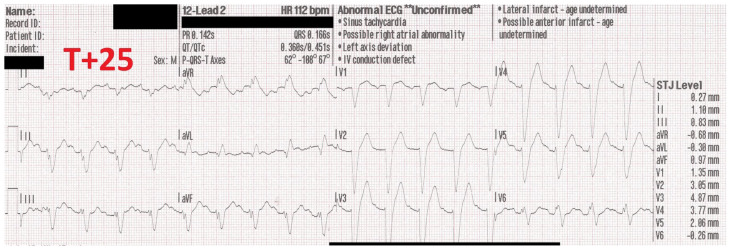
Smith S. Sudden Chest pain and SOB with a Wide Complex Tachycardia. In: Dr. Smith’s ECG Blog. CC BY-NC. https://drsmithsecgblog.com/sudden-chest-pain-and-sob-with-wide/

**Figure:**
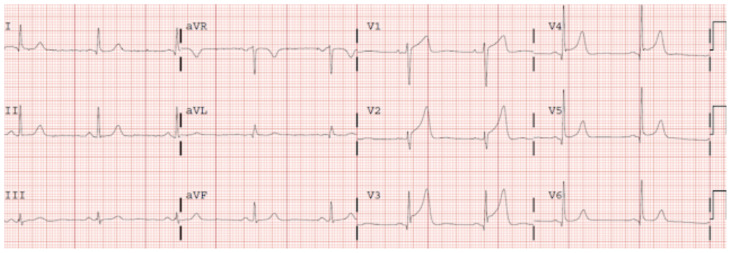
Smith S. Terminal QRS distortion not found in any ECG of Early Repolarization. In: Dr. Smith’s ECG Blog. CC BY-NC. https://drsmithsecgblog.com/paper-published-terminal-qrs-distortion/

**Figure:**
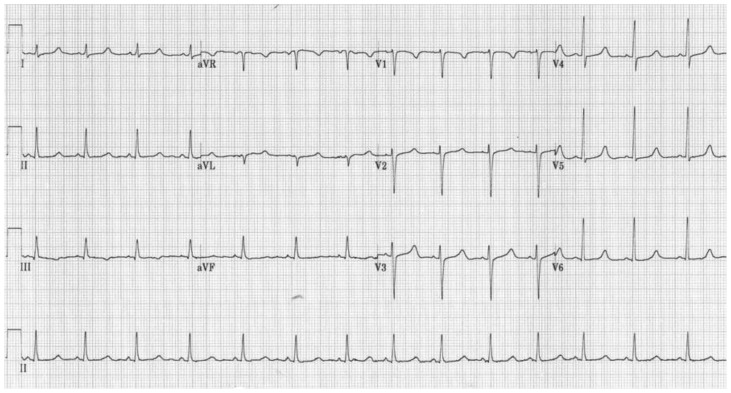
Burns E, Buttner R. Example 1, Hypocalcaemia. In: Life in the Fast Lane. CC BY-NC-SA. https://litfl.com/hypocalcaemia-ecg-library/

**Figure:**
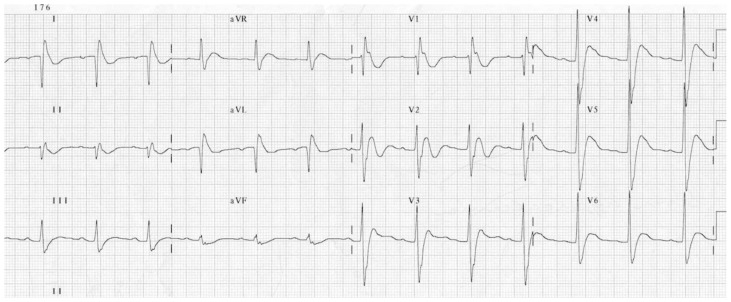
Burns E, Buttner R. Example 3, Hypercalcaemia. In: Life in the Fast Lane. CC BY-NC-SA. https://litfl.com/hypercalcaemia-ecg-library/

**Figure:**
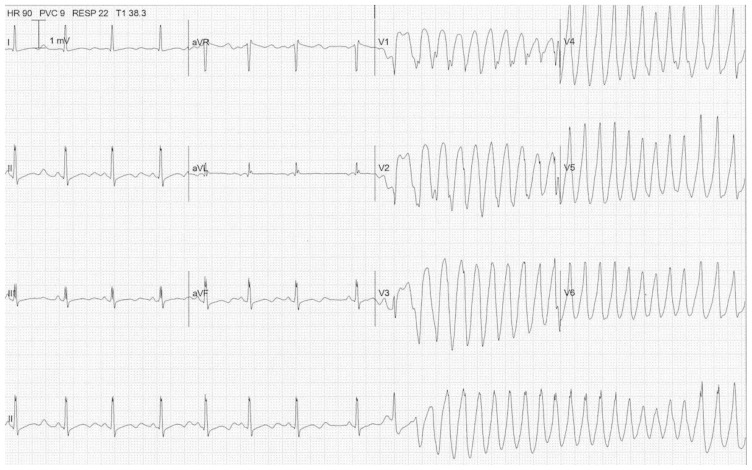
Burns E, Buttner R. Hypokalemia causing Torsades de Pointes. In: Life in the Fast Lane. CC BY-NC-SA. https://litfl.com/hypokalaemia-ecg-library/

**Figure:**
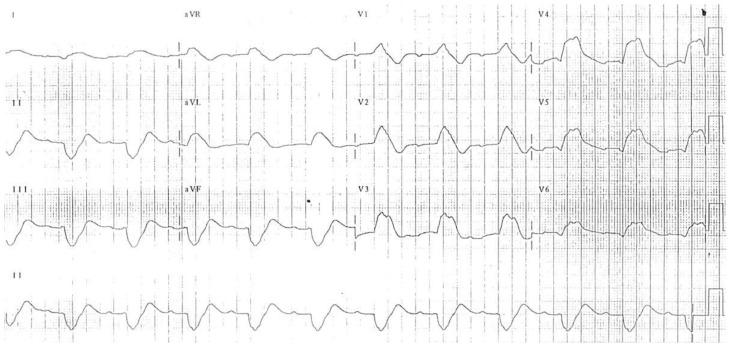
Burns E, Buttner R. Hyperkaelemia. In: Life in the Fast Lane. CC BY-NC-SA. https://litfl.com/hyperkalaemia-ecg-library/

**Figure:**
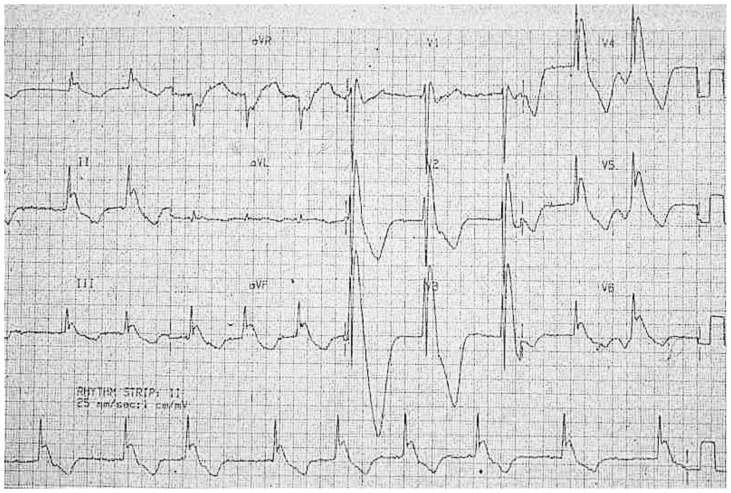
Burns E, Buttner R. Osborn Waves. In: Life in the Fast Lane. CC BY-NC-SA. https://litfl.com/hypothermia-ecg-library/

**Figure:**
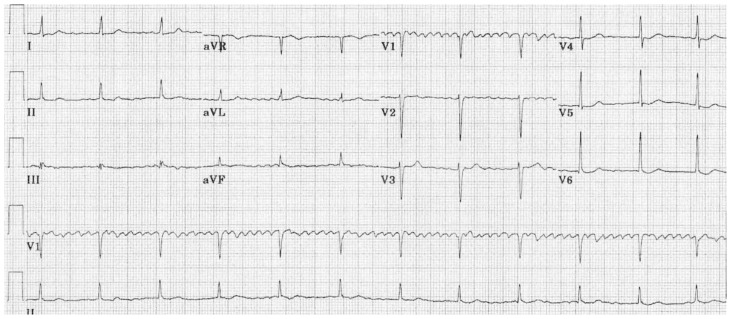
Burns E, Buttner R. Regularized atrial fibrillation (AF). In: Life in the Fast Lane. CC BY-NC-SA. https://litfl.com/digoxin-toxicity-ecg-library/

**Figure:**
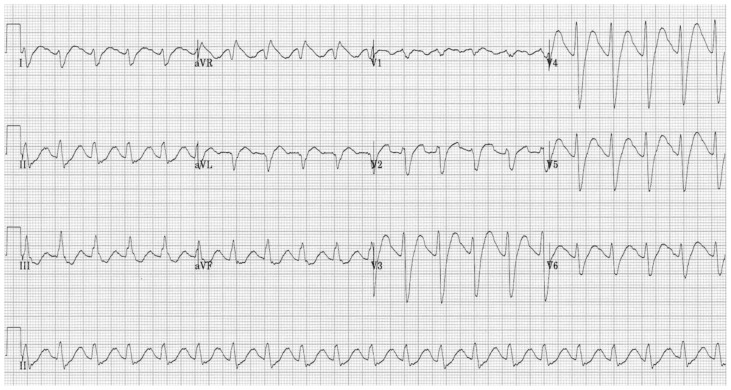
Burns E, Buttner R. tricyclic antidepressants (TCA) Toxicity. In: Life in the Fast Lane. CC BY-NC-SA. https://litfl.com/tricyclic-overdose-sodium-channel-blocker-toxicity/

**Figure:**
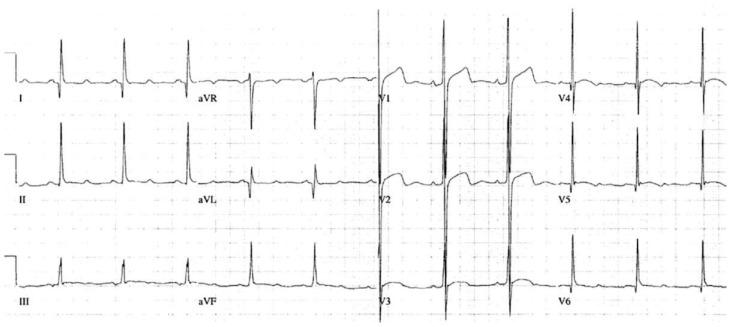
Burns E, Buttner R. HCM showing features of asymmetrical septal hypertrophy. In: Life in the Fast Lane. CC BY-NC-SA. https://litfl.com/hypertrophic-cardiomyopathy-hcm-ecg-library/

**Figure:**
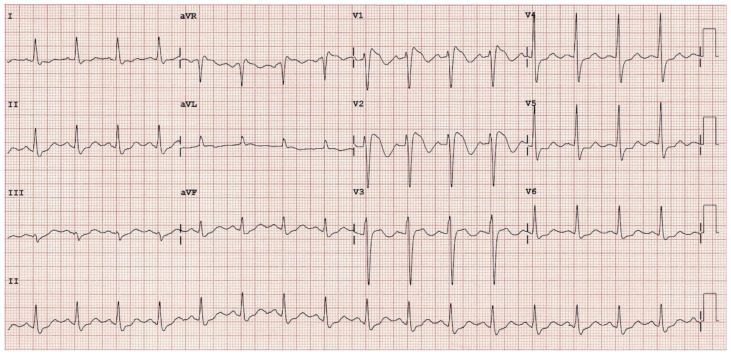
Larkin J, Cadogan M, Buttner R. Brugada Syndrome. In: Life in the Fast Lane. CC BY-NC-SA. https://litfl.com/brugada-syndrome-ecg-library

**Figure:**
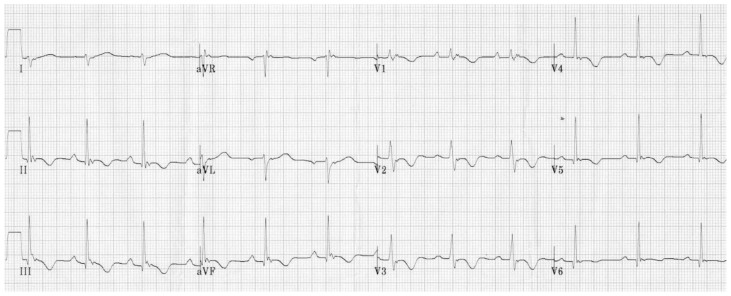
Buttner R, Burns E. Arrhythmogenic Right Ventricular Dysplasia, Example 1. In: Life in the Fast Lane. CC BY-NC-SA. https://litfl.com/arrhythmogenic-right-ventricular-dysplasia-arvd/

**Figure:**
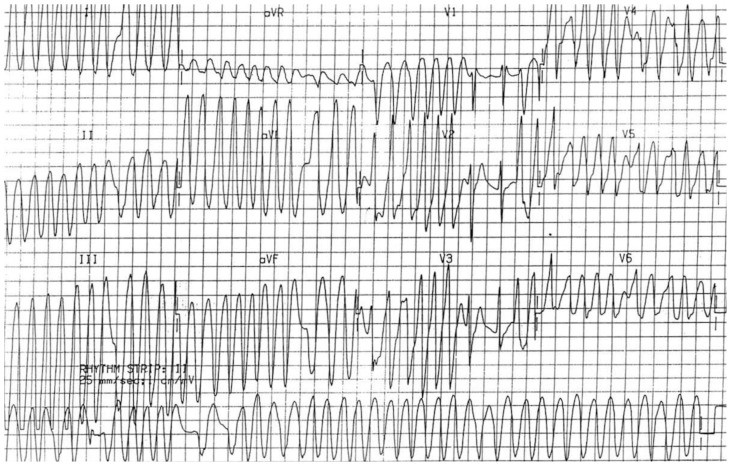
Buttner R. Atrial fibrillation/flutter in pre-excitation. Example 2. In: Life in the Fast Lane. CC BY-NC-SA. https://litfl.com/atrial-fibrillation-in-pre-excitation/

**Figure:**
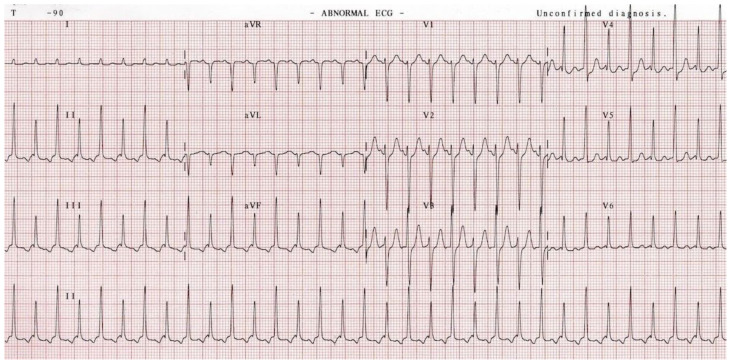
Burns E, Buttner R. supraventricular tachycardia (SVT) with QRS alternans. In: Life in the Fast Lane. CC BY-NC-SA. https://litfl.com/supraventricular-tachycardia-svt-ecg-library/

**Figure:**
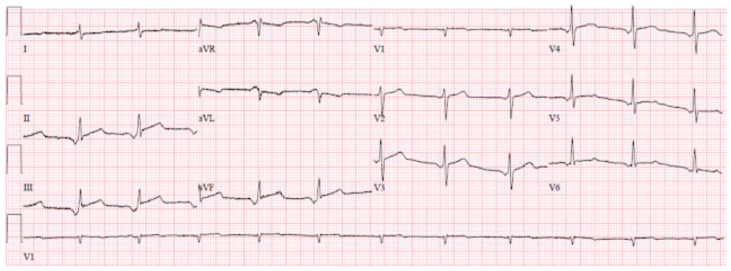
Meyers P. A man in his sixties with chest pain. In: Dr. Smith’s ECG Blog. CC BY-NC. https://drsmithsecgblog.com/a-man-in-his-sixties-with-chest-pain/

**Figure:**
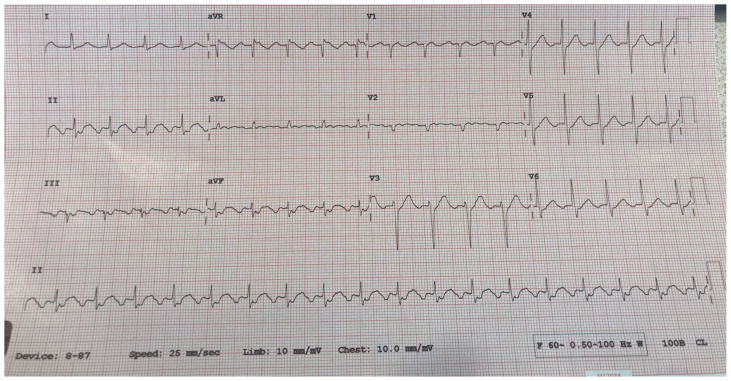
Meyers P. Global ST depression with ST elevation in a ventricular repolarization (VR); what is the cause? In: Dr. Smith’s ECG Blog. CC BY-NC. https://drsmithsecgblog.com/global-st-depression-with-st-elevation/

**Figure:**
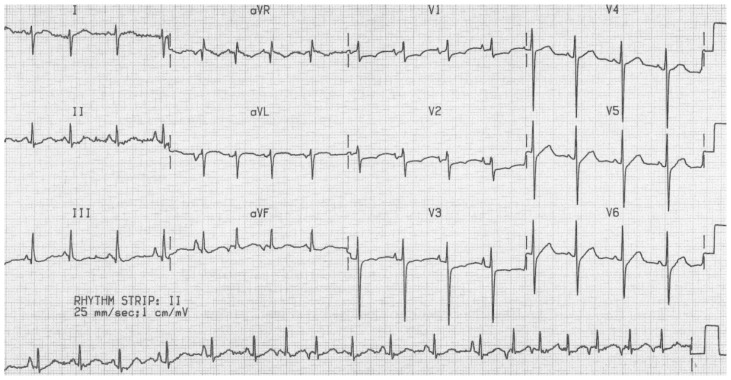
Burns E. Buttner R. Multifocal atrial tachycardia (MAT) with additional features of COPD. In: Life in the Fast Lane. CC BY-NC-SA. https://litfl.com/multifocal-atrial-tachycardia-mat-ecg-library/

**Figure:**
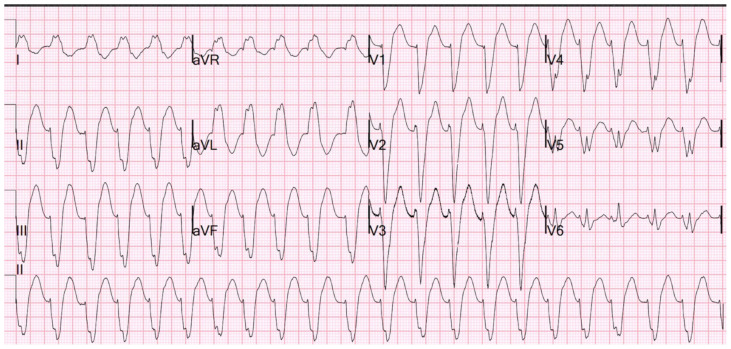
Smith S. Ventricular fibrillation, implantable cardioverter-defibrillator (ICD), left bundle branch block (LBBB), QRS of 210 ms, Positive Smith Modified Sgarbossa Criteria, and Pacemaker-Mediated Tachycardia. In: Dr. Smith’s ECG Blog. CC BY-NC. https://drsmithsecgblog.com/ventricular-fibrillation-icd-lbbb-qrs/

**Figure:**
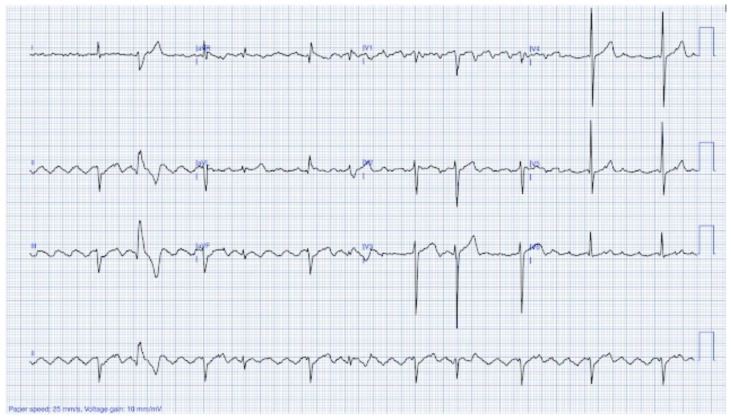
Frick W. And now for something completely different (a very difficult electrophysiology case). In: Dr. Smith’s ECG Blog. CC BY-NC. https://drsmithsecgblog.com/and-now-for-something-completely/

**Figure:**
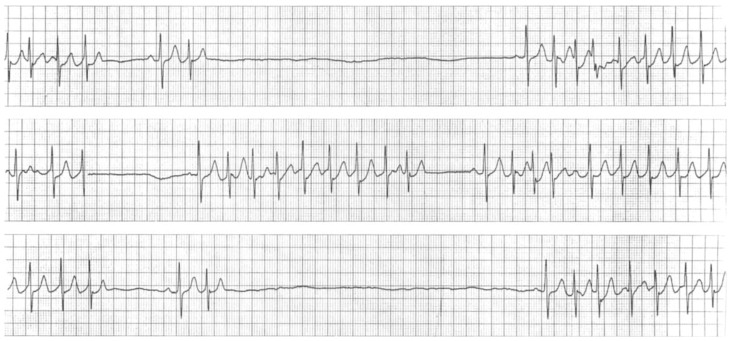
Burns E, Buttner R. Bradycardia-tachycardia syndrome. In: Life in the Fast Lane. CC BY-NC-SA. https://litfl.com/sinus-node-dysfunction-sick-sinus-syndrome/

**Figure:**
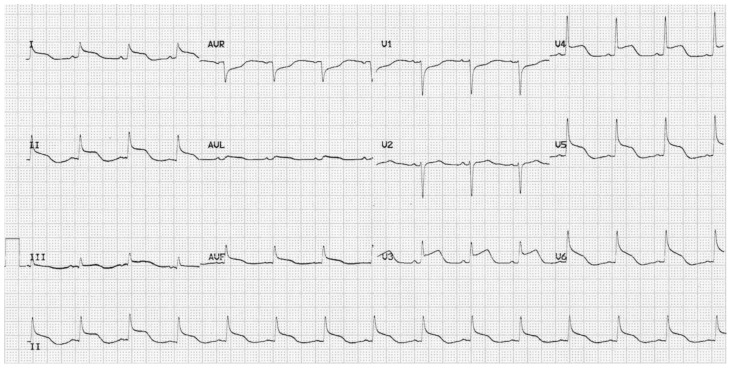
Cadogan M, Buttner R. Traumatic Brain Injury. In: Life in the Fast Lane. CC BY-NC-SA. https://litfl.com/raised-intracranial-pressure-ecg-library/

**Figure:**
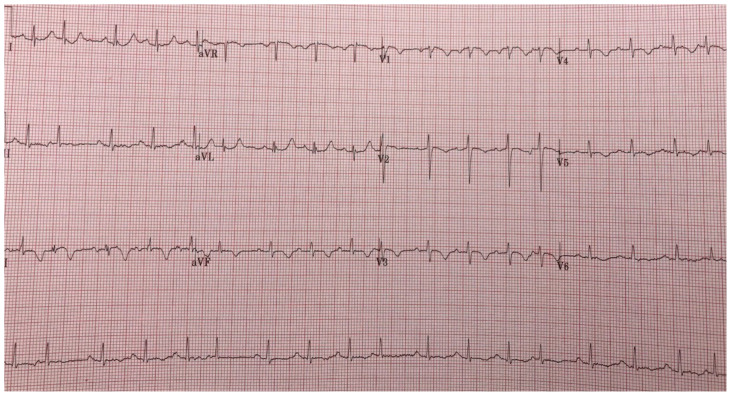
Meyers P. An elderly woman transferred to you for chest pain, shortness of breath, and positive troponin;does she need the cath lab now? In: Dr. Smith’s ECG Blog. CC BY-NC. https://drsmithsecgblog.com/an-elderly-woman-transferred-to-you-for/

**Figure:**
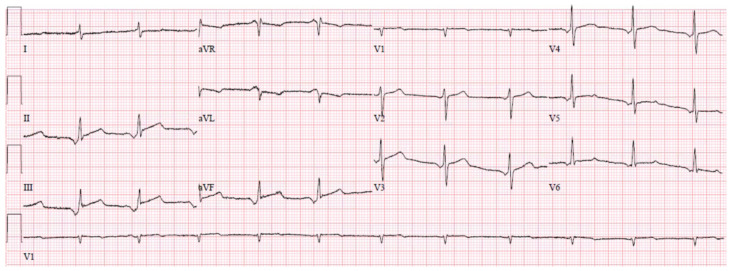
Meyers P. A man in his sixties with chest pain. In: Dr. Smith’s ECG Blog. CC BY-NC. https://drsmithsecgblog.com/a-man-in-his-sixties-with-chest-pain/

**Figure:**
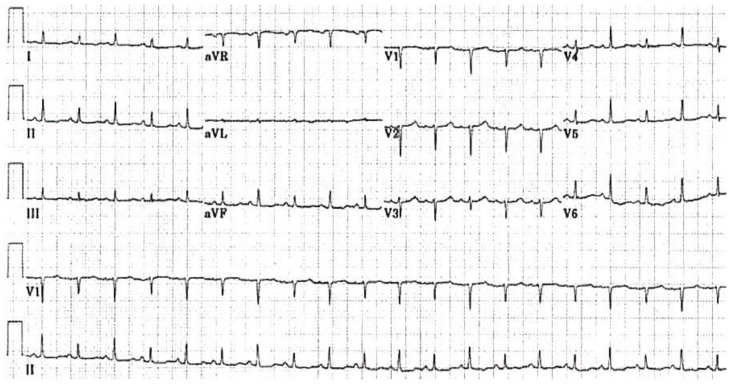
Burns E, Buttner R. Massive Pericardial Effusion. In: Life in the Fast Lane. CC BY-NC-SA. https://litfl.com/low-qrs-voltage-ecg-library/

**Figure:**
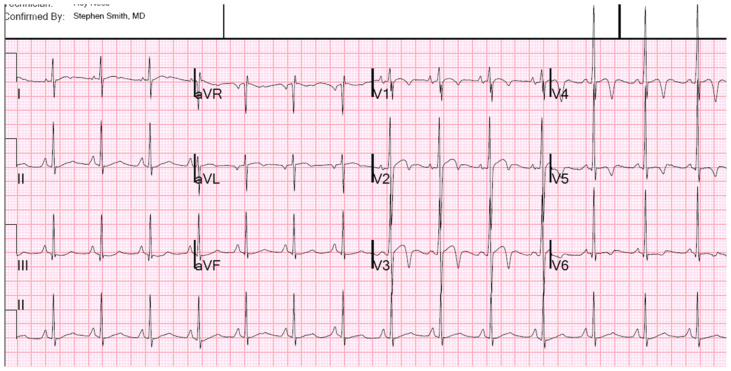
Smith S. Young African American Male with Atypical Stabbing Chest Pain. In: Dr. Smith’s ECG Blog. CC BY-NC. https://drsmithsecgblog.com/young-african-american-male-with/

**Figure:**
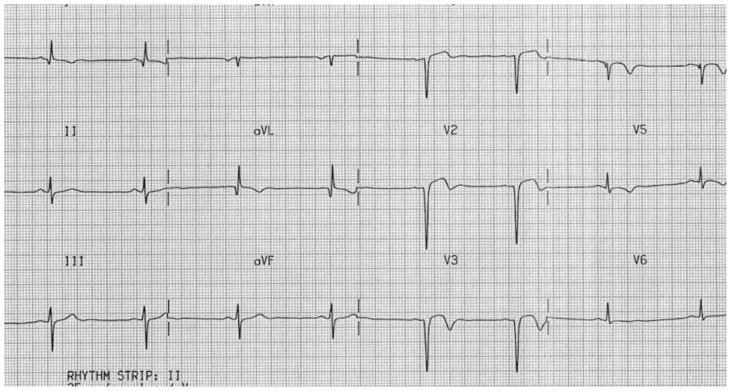
Burns E, Buttner R. Anterior Left Ventricular Aneurysm. In: Life in the Fast Lane. CC BY-NC-SA. https://litfl.com/left-ventricular-aneursym-ecg-library/

**Figure:**
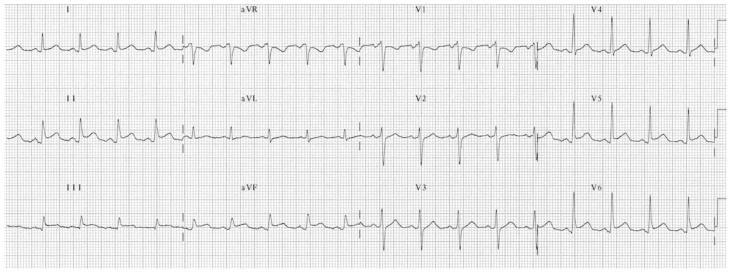
Burns E, Buttner R. Pericarditis. In: Life in the Fast Lane. CC BY-NC-SA. https://litfl.com/pericarditis-ecg-library/

**Figure:**
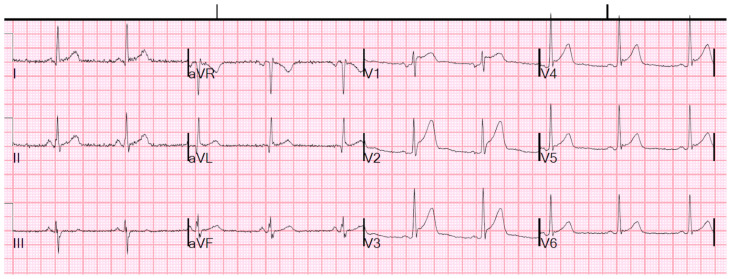
Smith S. A middle-aged male with chest pain. In: Dr. Smith’s ECG Blog. CC BY-NC. https://drsmithsecgblog.com/a-middle-aged-male-with-chest-pain/

**Figure:**
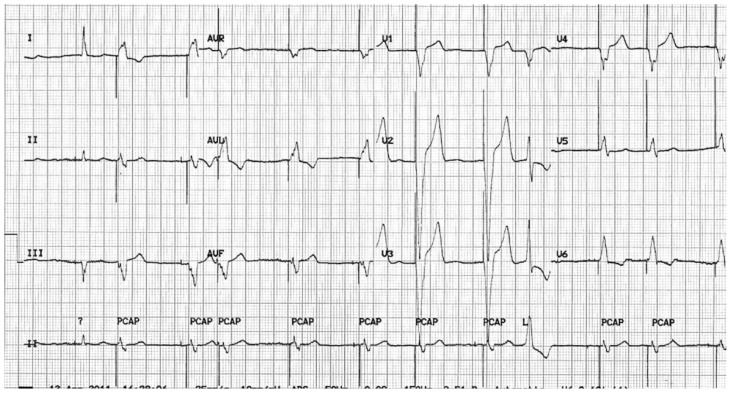
Burns E, Buttner R. Pacemaker Rhythms-Normal Patterns. In: Life in the Fast Lane. CC BY-NC-SA. https://litfl.com/pacemaker-rhythms-normal-patterns/

**Figure:**
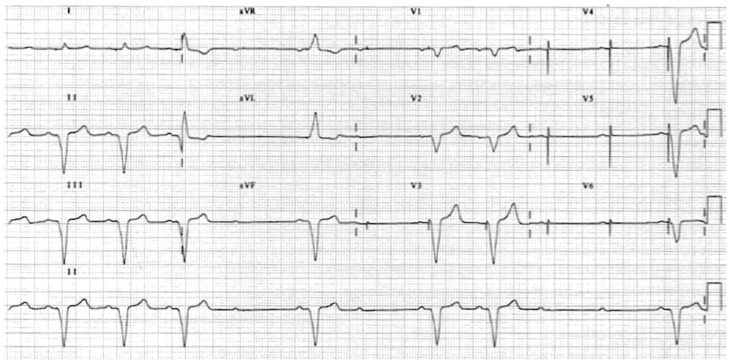
Burns E, Buttner R. Pacing failure. In: Life in the Fast Lane. CC BY-NC-SA. https://litfl.com/pacemaker-malfunction-ecg-library/

**Figure:**
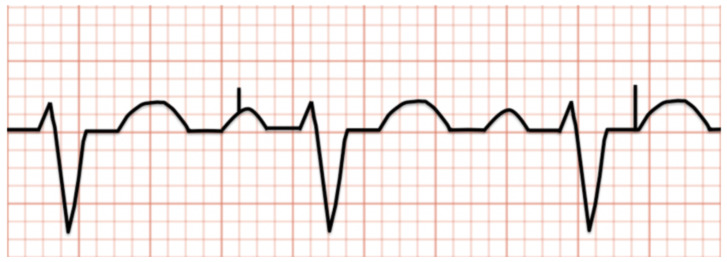
Palma J. Atrial impulse is inappropriately sent during native atrial depolarization. In: emDocs. CC BY-NC-SA. https://www.emdocs.net/em3am-pacemaker-aicd-complications/

**Figure:**
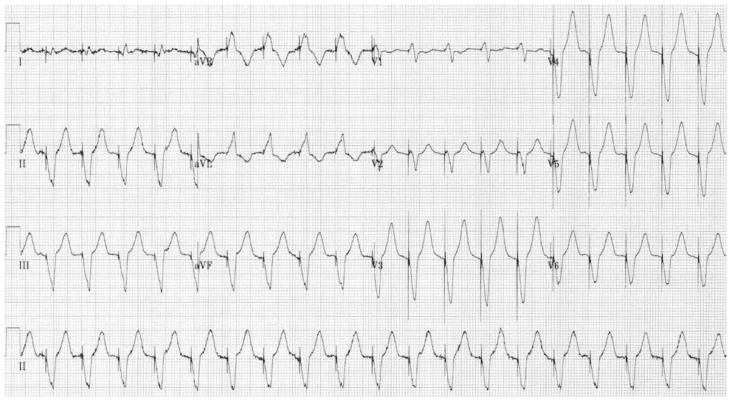
Burns E, Buttner R. Rapid ventricular pacing. In: Life in the Fast Lane. CC BY-NC-SA. https://litfl.com/pacemaker-malfunction-ecg-library/

**Figure:**
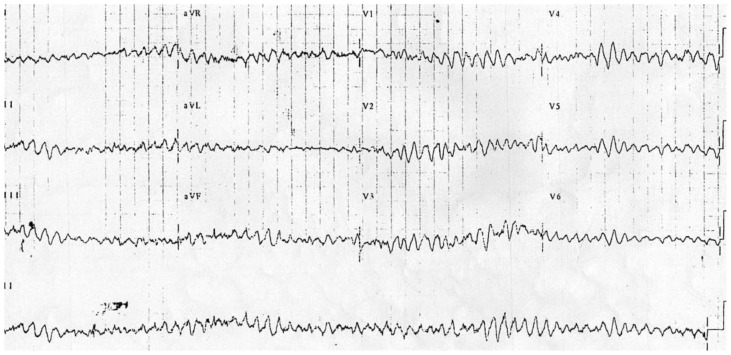
Burns E, Buttner R. Ventricular Fibrillation, Example 3. In: Life in the Fast Lane. CC BY-NC-SA. https://litfl.com/ventricular-fibrillation-vf-ecg-library/

**Figure:**
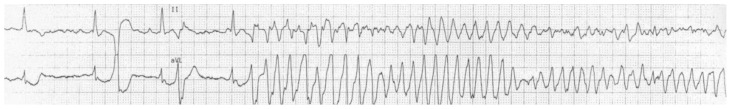
Burns E, Buttner R. Torsades de Pointes. In: Life in the Fast Lane. CC BY-NC-SA. https://litfl.com/polymorphic-vt-and-torsades-de-pointes-tdp

**Figure:**
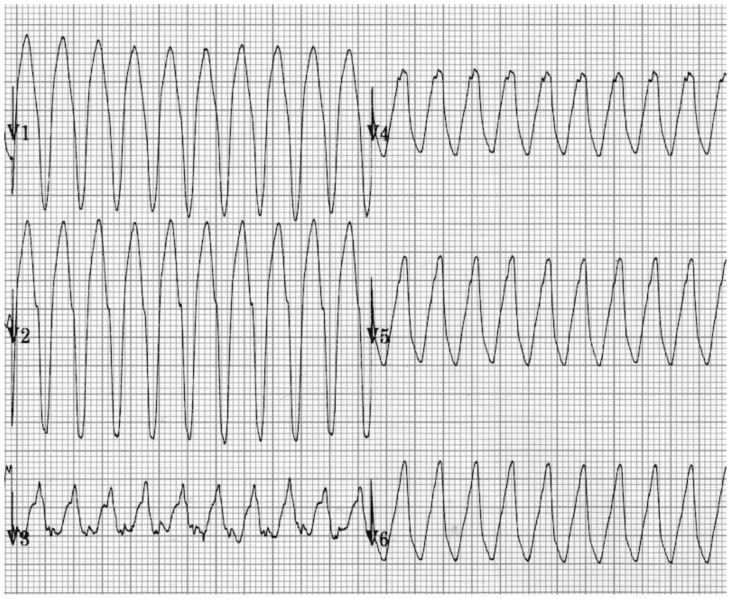
Burns E, Buttner R. Positive concordance. In: Life in the Fast Lane. CC BY-NC-SA. https://litfl.com/ventricular-tachycardia-monomorphic-ecg-library/

**Figure:**
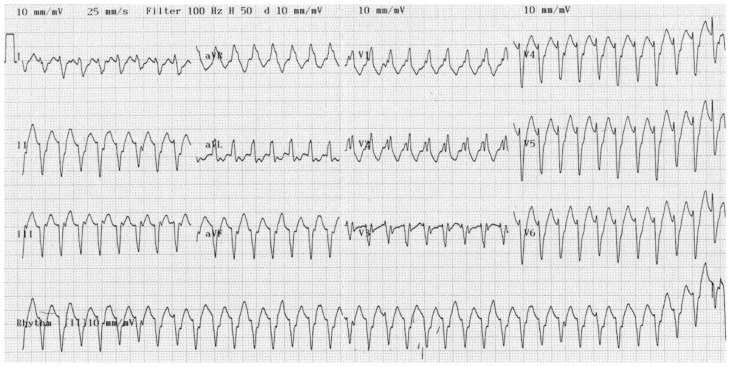
Burns E, Buttner R. Monomorphic VT. In: Life in the Fast Lane. CC BY-NC-SA. https://litfl.com/vt-versus-svt-ecg-library/

**Figure:**
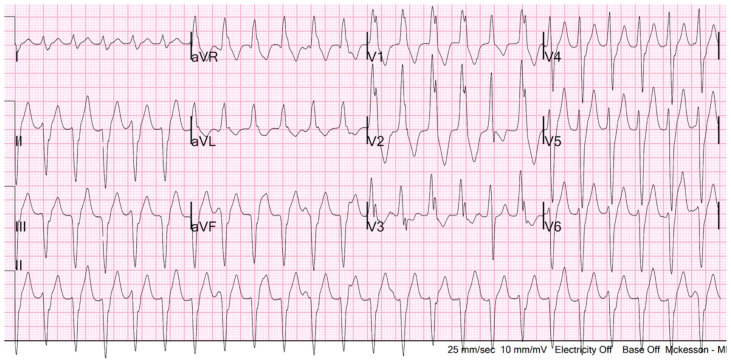
Smith S. A 50-something with Regular Wide Complex Tachycardia. What to do if electrical cardioversion does not work? In: Dr. Smith’s ECG Blog. CC BY-NC. https://drsmithsecgblog.com/a-50-something-with-regular-wide/

**Figure:**
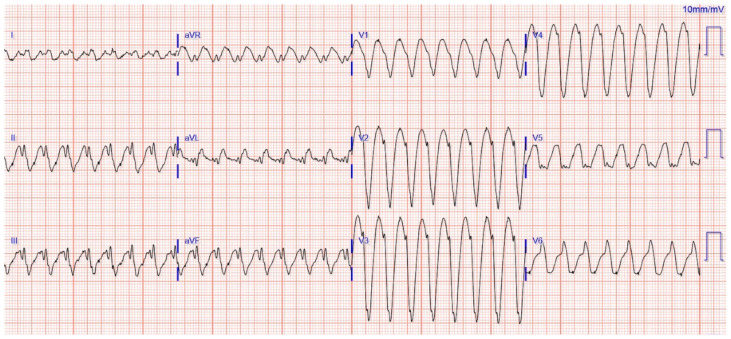
Walsh B. Wide-complex tachycardia that didn’t follow the rules. In: Dr. Smith’s ECG Blog. CC BY-NC. https://drsmithsecgblog.com/wide-complex-tachycardia-that-didnt/

**Figure:**
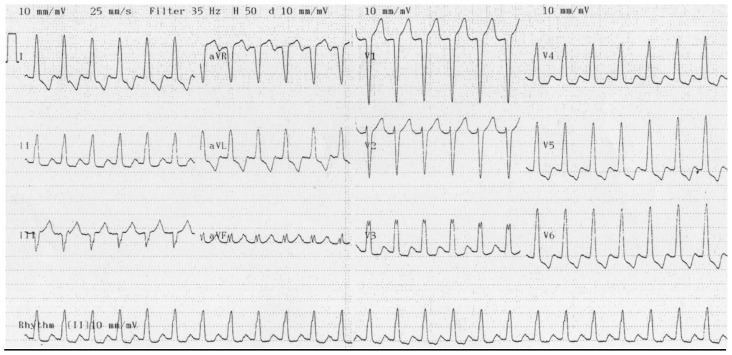
Burns E, Buttner R. Atrioventricular-nodal re-entry tachycardia (AVNRT) with LBBB. In: Life in the Fast Lane. CC BY-NC-SA. https://litfl.com/vt-versus-svt-ecg-library/

**Figure:**
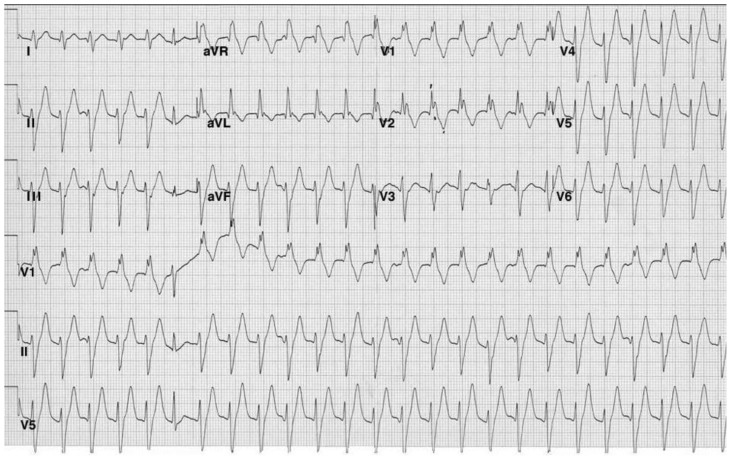
Burns E, Cadogan M. Idiopathic Fascicular VT. In: Life in the Fast Lane. CC BY-NC-SA. https://litfl.com/idiopathic-fascicular-left-ventricular-tachycardia/

## Appendix A Overview of Logistics for System Automatization

### System Overview

A Google Drive folder was created that contained an images folder housing the ECG images, the Google Form for resident answers, the Google Sheet compiling resident answers, and the Google Sheet that contained emails, the brief clinical case presentation and relevant links, and the dates for the emails to be sent.

### Emails

The brief clinical case (i.e. 30-year-old presenting with palpitations) was sent out automatically to residents on conference days with a picture of the ECG for interpretation as well as a link to their interpretation using Google Forms. The emails were scheduled to be sent out on the morning of conference days every week.

### Resident Interpretations

The residents were able to anonymously submit their interpretation to the email using a link to a Google Form. The Google Form contains the week number listed that residents do not change, but they provide a brief interpretation (i.e. MAT) that is free texted. These answers are compiled automatically onto a Google Sheets document that lists the week number of the case and the resident interpretations. This allows for continual monitoring of resident answers to identify topics with which residents may find difficult.

### Answers and Topic Pages

In the first year of implementation, a follow-up email was sent the next day with the answer and explanation. In the second year of implementation, a website was created using Google Sites that contained all of the topic pages within the curriculum, as well as additional study material including a quiz. There was then no follow-up email with answers as the initial email contained a link to the topic page that was the answer for the ECG that week.

## Appendix D Weekly EKG Cases

**Week 1:** 65-year-old presenting with chest pain (CP).

Answer submission: Insert program-specific Google Forms link

Answer page: https://www.ccagem.com/ekg-epistemology

**Week 2:** 45-year-old presenting with dizziness.

Answer submission: Insert program-specific Google Forms link

Answer page: https://www.ccagem.com/ekg-epistemology

**Week 3:** 25-year-old presenting with nausea and vomiting.

Answer submission: Insert program-specific Google Forms link

Answer page: https://www.ccagem.com/ekg-epistemology

**Week 4:** 65-year-old presenting with syncope.

Answer submission: Insert program-specific Google Forms link

Answer page: https://www.ccagem.com/ekg-epistemology

**Week 5:** 70-year-old presenting with vomiting.

Answer submission: Insert program-specific Google Forms link

Answer page: https://www.ccagem.com/ekg-epistemology

**Week 6:** 35yo PMHx asthma presenting with shortness of breath (SOB).

Answer submission: Insert program-specific Google Forms link

Answer page: https://www.ccagem.com/ekg-epistemology

**Week 7:** 30yo presenting with fasciculations, just had surgery.

Answer submission: Insert program-specific Google Forms link

Answer page: https://www.ccagem.com/ekg-epistemology

**Week 8:** 45yo presenting with chest pain.

Answer submission: Insert program-specific Google Forms link

Answer page: https://www.ccagem.com/ekg-epistemology

**Week 9:** 30yo presenting with SOB.

Answer submission: Insert program-specific Google Forms link

Answer page: https://www.ccagem.com/ekg-epistemology

**Week 10:** 45yo presenting with CP.

Answer submission: Insert program-specific Google Forms link

Answer page: https://www.ccagem.com/ekg-epistemology

**Week 11:** 20yo presenting with palpitations.

Answer submission: Insert program-specific Google Forms link

Answer page: https://www.ccagem.com/ekg-epistemology

**Week 12:** 20yo presenting with palpitations.

Answer submission: Insert program-specific Google Forms link

Answer page: https://www.ccagem.com/ekg-epistemology

**Week 13:** 1yo presenting for vomiting/diarrhea.

Answer submission: Insert program-specific Google Forms link

Answer page: https://www.ccagem.com/ekg-epistemology

**Week 14:** 60yo presenting for 15hours of chest pain (CP).

Answer submission: Insert program-specific Google Forms link

Answer page: https://www.ccagem.com/ekg-epistemology/week-14-26

**Week 15:** 45yo presenting with shock, no history.

Answer submission: Insert program-specific Google Forms link

Answer page: https://www.ccagem.com/ekg-epistemology/week-14-26

**Week 16:** 45yo presenting with CP ~2hours ago that resolved.

Answer submission: Insert program-specific Google Forms link

Answer page: https://www.ccagem.com/ekg-epistemology/week-14-26

**Week 17:** 55yo presenting with 30min of CP.

Answer submission: Insert program-specific Google Forms link

Answer page: https://www.ccagem.com/ekg-epistemology/week-14-26

**Week 18:** 60yo presenting for 45min of CP.

Answer submission: Insert program-specific Google Forms link

Answer page: https://www.ccagem.com/ekg-epistemology/week-14-26

**Week 19:** 45yo presenting status post return of spontaneous circulation (ROSC).

Answer submission: Insert program-specific Google Forms link

Answer page: https://www.ccagem.com/ekg-epistemology/week-14-26

**Week 20:** 35yo presenting with 20 min of CP (they were here getting a pop anyways).

Answer submission: Insert program-specific Google Forms link

Answer page: https://www.ccagem.com/ekg-epistemology/week-14-26

**Week 21:** 80yo presenting with exertional SOB.

Answer submission: Insert program-specific Google Forms link

Answer page: https://www.ccagem.com/ekg-epistemology/week-14-26

**Week 22:** 60yo presenting with shock after reporting CP.

Answer submission: Insert program-specific Google Forms link

Answer page: https://www.ccagem.com/ekg-epistemology/week-14-26

**Week 23:** 40yo presenting with SOB.

Answer submission: Insert program-specific Google Forms link

Answer page: https://www.ccagem.com/ekg-epistemology/week-14-26

**Week 24:** 70yo presenting with SOB.

Answer submission: Insert program-specific Google Forms link

Answer page: https://www.ccagem.com/ekg-epistemology/week-14-26

**Week 25:** 35yo presenting with sepsis.

Answer submission: Insert program-specific Google Forms link

Answer page: https://www.ccagem.com/ekg-epistemology/week-14-26

**Week 26:** 50 presenting with CP.

Answer submission: Insert program-specific Google Forms link

Answer page: https://www.ccagem.com/ekg-epistemology/week-14-26

**Week 27:** 60 presenting with CP and near syncope.

Answer submission: Insert program-specific Google Forms link

Answer page: https://www.ccagem.com/ekg-epistemology/week-27-39

**Week 28:** 55yo presenting with syncope.

Answer submission: Insert program-specific Google Forms link

Answer page: https://www.ccagem.com/ekg-epistemology/week-27-39

**Week 29:** 35yo presenting with palpitations.

Answer submission: Insert program-specific Google Forms link

Answer page: https://www.ccagem.com/ekg-epistemology/week-27-39

**Week 30:** 40yo presenting with large vessel occlusion (LVO) stroke.

Answer submission: Insert program-specific Google Forms link

Answer page: https://www.ccagem.com/ekg-epistemology/week-27-39

**Week 31:** 35yo presenting with palpitations; do you send troponin?

Answer submission: Insert program-specific Google Forms link

Answer page: https://www.ccagem.com/ekg-epistemology/week-27-39

**Week 32:** 55yo presenting with CP.

Answer submission: Insert program-specific Google Forms link

Answer page: https://www.ccagem.com/ekg-epistemology/week-27-39

**Week 33:** 60yo presenting with SOB.

Answer submission: Insert program-specific Google Forms link

Answer page: https://www.ccagem.com/ekg-epistemology/week-27-39

**Week 34:** 60 presenting with SOB after CP 1 week ago.

Answer submission: Insert program-specific Google Forms link

Answer page: https://www.ccagem.com/ekg-epistemology/week-27-39

**Week 35:** 55yo presenting with CP.

Answer submission: Insert program-specific Google Forms link

Answer page: https://www.ccagem.com/ekg-epistemology/week-27-39

**Week 36:** 35yo presenting with CP.

Answer submission: Insert program-specific Google Forms link

Answer page: https://www.ccagem.com/ekg-epistemology/week-27-39

**Week 37:** 30yo presenting for fatigue and muscle spasms after neck surgery.

Answer submission: Insert program-specific Google Forms link

Answer page: https://www.ccagem.com/ekg-epistemology/week-27-39

**Week 38:** 70yo presenting with fatigue and confusion.

Answer submission: Insert program-specific Google Forms link

Answer page: https://www.ccagem.com/ekg-epistemology/week-27-39

**Week 39:** 40yo presenting with...crap just look at the EKG.

Answer submission: Insert program-specific Google Forms link

Answer page: https://www.ccagem.com/ekg-epistemology/week-27-39

**Week 40:** 35yo presenting dehydrated and confused.

Answer submission: Insert program-specific Google Forms link

Answer page: https://www.ccagem.com/ekg-epistemology/week-40-52

**Week 41:** 55yo presenting as found down outside, altered mental status (AMS).

Answer submission: Insert program-specific Google Forms link

Answer page: https://www.ccagem.com/ekg-epistemology/week-40-52

**Week 42:** 70yo presenting for vomiting and weakness.

Answer submission: Insert program-specific Google Forms link

Answer page: https://www.ccagem.com/ekg-epistemology/week-40-52

**Week 43:** 18yo presenting with vomiting.

Answer submission: Insert program-specific Google Forms link

Answer page: https://www.ccagem.com/ekg-epistemology/week-40-52

**Week 44:** 18yo presenting with syncope.

Answer submission: Insert program-specific Google Forms link

Answer page: https://www.ccagem.com/ekg-epistemology/week-40-52

**Week 45:** 30yo presenting after ventricular tachycardia (VT) arrest s/p ROSC.

Answer submission: Insert program-specific Google Forms link

Answer page: https://www.ccagem.com/ekg-epistemology/week-40-52

**Week 46:** 35yo Italian presenting after syncope.

Answer submission: Insert program-specific Google Forms link

Answer page: https://www.ccagem.com/ekg-epistemology/week-40-52

**Week 47:** 30yo presenting in shock.

Answer submission: Insert program-specific Google Forms link

Answer page: https://www.ccagem.com/ekg-epistemology/week-40-52

**Week 48:** 35yo presenting with dizziness.

Answer submission: Insert program-specific Google Forms link

Answer page: https://www.ccagem.com/ekg-epistemology/week-40-52

**Week 49:** 60yo presenting with congestive heart failure (CHF) exacerbation.

Answer submission: Insert program-specific Google Forms link

Answer page: https://www.ccagem.com/ekg-epistemology/week-40-52

**Week 50:** 60yo presenting with shortness of breath.

Answer submission: Insert program-specific Google Forms link

Answer page: https://www.ccagem.com/ekg-epistemology/week-40-52

**Week 51:** 60yo presenting with shortness of breath.

Answer submission: Insert program-specific Google Forms link

Answer page: https://www.ccagem.com/ekg-epistemology/week-40-52

**Week 52:** 60 presenting with palpitations, CP, and SOB.

Answer submission: Insert program-specific Google Forms link

Answer page: https://www.ccagem.com/ekg-epistemology/week-40-52

**Week 53:** 50yo presenting with “my afib.”

Answer submission: Insert program-specific Google Forms link

Answer page: https://www.ccagem.com/ekg-epistemology/week-53-65

**Week 54:** 70yo hx coronary artery disease (CAD) presenting after syncope; nurse records this strip.

Answer submission: Insert program-specific Google Forms link

Answer page: https://www.ccagem.com/ekg-epistemology/week-53-65

**Week 55:** 55yo presenting with headache, altered mental status and bilateral leg weakness.

Answer submission: Insert program-specific Google Forms link

Answer page: https://www.ccagem.com/ekg-epistemology/week-53-65

**Week 56:** 40yo presenting with respiratory distress.

Answer submission: Insert program-specific Google Forms link

Answer page: https://www.ccagem.com/ekg-epistemology/week-53-65

**Week 57:** 30yo presenting with vomiting and diarrhea with tachycardia.

Answer submission: Insert program-specific Google Forms link

Answer page: https://www.ccagem.com/ekg-epistemology/week-53-65

**Week 58:** 35yo presenting with syncope.

Answer submission: Insert program-specific Google Forms link

Answer page: https://www.ccagem.com/ekg-epistemology/week-53-65

**Week 59:** 25yo presenting with CP.

Answer submission: Insert program-specific Google Forms link

Answer page: https://www.ccagem.com/ekg-epistemology/week-53-65

**Week 60:** 45yo presenting with SOB and lower extremity (LE) edema.

Answer submission: Insert program-specific Google Forms link

Answer page: https://www.ccagem.com/ekg-epistemology/week-53-65

**Week 61:** 45yo presenting with CP.

Answer submission: Insert program-specific Google Forms link

Answer page: https://www.ccagem.com/ekg-epistemology/week-53-65

**Week 62:** 35yo presenting with CP.

Answer submission: Insert program-specific Google Forms link

Answer page: https://www.ccagem.com/ekg-epistemology/week-53-65

**Week 63:** 60yo presenting with SOB.

Answer submission: Insert program-specific Google Forms link

Answer page: https://www.ccagem.com/ekg-epistemology/week-53-65

**Week 64:** 60yo presenting with syncope.

Answer submission: Insert program-specific Google Forms link

Answer page: https://www.ccagem.com/ekg-epistemology/week-53-65

**Week 65:** 60yo presenting with nausea and vomiting.

Answer submission: Insert program-specific Google Forms link

Answer page: https://www.ccagem.com/ekg-epistemology/week-53-65

**Week 66:** 90yo presenting with tachycardia.

Answer submission: Insert program-specific Google Forms link

Answer page: https://www.ccagem.com/ekg-epistemology/week-66-74

**Week 67:** Nursing asks you to see a patient in this room and hands you this EKG.

Answer submission: Insert program-specific Google Forms link

Answer page: https://www.ccagem.com/ekg-epistemology/week-66-74

**Week 68:** 35yo psych patient with vomiting.

Answer submission: Insert program-specific Google Forms link

Answer page: https://www.ccagem.com/ekg-epistemology/week-66-74

**Week 69:** 40yo presenting with palpitations.

Answer submission: Insert program-specific Google Forms link

Answer page: https://www.ccagem.com/ekg-epistemology/week-66-74

**Week 70:** 40yo presenting with palpitations.

Answer submission: Insert program-specific Google Forms link

Answer page: https://www.ccagem.com/ekg-epistemology/week-66-74

**Week 71:** 35yo presenting with palpitations.

Answer submission: Insert program-specific Google Forms link

Answer page: https://www.ccagem.com/ekg-epistemology/week-66-74

**Week 72:** 50yo presenting with SOB.

Answer submission: Insert program-specific Google Forms link

Answer page: https://www.ccagem.com/ekg-epistemology/week-66-74

**Week 73:** 45yo presenting with chest pain and palpitations.

Answer submission: Insert program-specific Google Forms link

Answer page: https://www.ccagem.com/ekg-epistemology/week-66-74

**Week 74:** 18yo presenting for palpitations.

Answer submission: Insert program-specific Google Forms link

Answer page: https://www.ccagem.com/ekg-epistemology/week-66-74

## References

[1] Rolskov Bojsen S, Räder SB, Holst AG, Kayser L, Ringsted C (2015). The acquisition and retention of ECG interpretation skills after a standardized web-based ECG tutorial-a randomised study. BMC Med Educ.

[2] Patocka C, Turner J, Wiseman J (2015). What adult electrocardiogram (ECG) diagnoses and/or findings do residents in emergency medicine need to know?. CJEM.

[3] Bilello LA, Pascheles C, Gurley K, Rappaport D, Chiu DT (2020). Getting to the heart of the issue: senior emergency resident electrocardiogram interpretation and its impact on quality assurance events. Clin Exp Emerg Med.

[4] Calixte D, Haynes NA, Robert M, Edmond C, Yan LD (2022). Online team-based electrocardiogram training in Haiti: evidence from the field. BMC Med Educ.

[5] Nilsson M, Östergren J, Fors U, Rickenlund A, Jorfeldt L (2012). Does individual learning styles influence the choice to use a web-based ECG learning programme in a blended learning setting?. BMC Med Educ.

[6] Ginde AA, Char DM (2003). Emergency medicine residency training in electrocardiogram interpretation. Acad Emerg Med.

[7] Raupach T, Harendza S, Anders S, Schuelper N, Brown J (2016). How can we improve teaching of ECG interpretation skills? Findings from a prospective randomised trial. J Electrocardiol.

[8] Viljoen CA, Millar RS, Manning K, Hoevelmann J, Burch VC (2021). Clinically contextualised ECG interpretation: the impact of prior clinical exposure and case vignettes on ECG diagnostic accuracy. BMC Med Educ.

[9] Pines JM, Perina DG, Brady WJ (2004). Electrocardiogram interpretation training and competency assessment in emergency medicine residency programs. Acad Emerg Med.

[10] Fent G, Gosai J, Purva M (2015). Teaching the interpretation of electrocardiograms: which method is best?. J Electrocardiol.

[11] Viljoen CA, Millar RS, Manning K, Burch VC (2020). Effectiveness of blended learning versus lectures alone on ECG analysis and interpretation by medical students. BMC Med Educ.

[12] Oh SY, Cook DA, Van Gerven PWM, Nicholson J, Fairbrother H (2022). Physician Training for Electrocardiogram Interpretation: A Systematic Review and Meta-Analysis. Acad Med.

[13] Klein AJ, Berlacher M, Doran JA, Corbelli J, Rothenberger SD, Berlacher K (2020). A Resident-Authored, Case-Based Electrocardiogram Email Curriculum for Internal Medicine Residents. MedEdPORTAL.

[14] Krasne S, Stevens CD, Kellman PJ, Niemann JT (2020). Mastering Electrocardiogram Interpretation Skills Through a Perceptual and Adaptive Learning Module. AEM Educ Train.

[15] Viljoen CA, Scott Millar R, Engel ME, Shelton M, Burch V (2019). Is computer-assisted instruction more effective than other educational methods in achieving ECG competence amongst medical students and residents? A systematic review and meta-analysis. BMJ Open.

[16] Monteiro S, Melvin L, Manolakos J, Patel A, Norman G (2017). Evaluating the effect of instruction and practice schedule on the acquisition of ECG interpretation skills. Perspect Med Educ.

[17] Burns WP, Hartman ND, Weygandt PL, Jones SC, Caretta-Weyer H, Moore KG (2019). Critical Electrocardiogram Curriculum: Setting the Standard for Flipped-Classroom EKG Instruction. West J Emerg Med.

[18] Beeson MS, Ankel F, Bhat R, Broder JS, Dimeo SP (2020). The 2019 Model of the Clinical Practice of Emergency Medicine. J Emerg Med.

[19] Salerno SM, Alguire PC, Waxman HS, American College of Physicians (2003). Training and competency evaluation for interpretation of 12-lead electrocardiograms: recommendations from the American College of Physicians. Ann Intern Med.

[20] Salerno SM, Alguire PC, Waxman HS (2003). Competency in interpretation of 12-lead electrocardiograms: a summary and appraisal of published evidence. Ann Intern Med.

[21] Young TP, Bailey CJ, Guptill M, Thorp AW, Thomas TL (2014). The flipped classroom: a modality for mixed asynchronous and synchronous learning in a residency program. West J Emerg Med.

[22] Pourmand A, Tanski M, Davis S, Shokoohi H, Lucas R, Zaver F (2015). Educational technology improves ECG interpretation of acute myocardial infarction among medical students and emergency medicine residents. West J Emerg Med.

[23] Liu SS, Zakaria S, Vaidya D, Srivastava MC (2017). Electrocardiogram training for residents: A curriculum based on Facebook and Twitter. J Electrocardiol.

[24] Burns E, Buttner R Pacemaker Rhythms-Normal Patterns. Life in the Fast Lane.

[25] Swaminathan A Pacemaker Basics.

[26] Cadogan M, Buttner R Sgarbossa Criteria. Life in the Fast Lane.

[27] Larkin J, Burrows C (2015). Interpretation. ECG of the Week.

