# Supplementary material for: Enhancing Emergency Medicine Resident Education: A Weekly Education Series to Augment Electrocardiogram Education
Source: J Educ Teach Emerg Med. 2026 Jan 31;11(1):C1–C89. doi: 10.5070/M5.52141 (PMC12880887; doi:10.5070/M5.52141)
Supplement: Supplementary file 1 [file 11-1-C1-AppendixB.pdf]

DO NOT CHANGE ANY LAYOUT OF THIS SPREADSHEET! ONLY CHANGE DATA!

Email Adresses	Date	Subject	Text	Image
	1/9/2025	Week 1	65 year old presenting with chest pain; htt	https://drive.google.com/file/d/1sw5QLsCu1BXMECo6hOL_Gx8iKDqiMRj8/preview
	1/16/2025	Week 2	45 year old presenting with dizziness; http	https://drive.google.com/file/d/1PJvtKeJGp6hwB1MFHCOmDyHM0KdkBBnQ/view?usp=sharing
	1/23/2025	Week 3	25 year old presenting with nausea and vo	https://drive.google.com/file/d/1D51z1BS2eUECUzVpJ0-HL-txN5VpSZyX/view?usp=sharing
	1/30/2025	Week 4	65 year old presenting with syncope; https	https://drive.google.com/file/d/1VHjahlu_sQ1VZ7L6DFl-fkl4pFkUgFtl/view?usp=sharing
	2/6/2025	Week 5	70 year old presenting with vomiting; https	https://drive.google.com/file/d/1aRF8KPta-Ib8SF9wmmHF6E_ci2Dtxs_4/view?usp=sharing
	2/13/2025	Week 6	35yo PMHx asthma presenting with SOB;	https://drive.google.com/file/d/1BQHMlRi4-d8tMie6P9kALCVgggK1EXRZ/view?usp=sharing
	2/20/2025	Week 7	30yo presenting with fasciculations, just ha	https://drive.google.com/file/d/1uf0xn_T486aiOZCHDtZR25tp4PXYas7M/view?usp=sharing
	2/27/2025	Week 8	45yo presenting with chest pain; https://fo	https://drive.google.com/file/d/1j51_AXnrRXoSaic4TtiBkLByAGs0K8ss/view?usp=sharing
	3/6/2025	Week 9	30yo presenting with SOB; https://forms.g	https://drive.google.com/file/d/1N-JEt-s-3sxtW7Gb6l1kNClBdT2ZiGG5/view?usp=sharing
	3/13/2025	Week 10	45yo presenting with CP; https://forms.gle	https://drive.google.com/file/d/165BTeWEYbHVSi1W4vhMhWRWH9qGwW0H5/view?usp=sharing
	3/20/2025	Week 11	20yo presenting with palpitations; https://fo	https://drive.google.com/file/d/1ylDXRtqhrKwa7aeXjZcZhSN23K0c_yi4/view?usp=sharing
	3/27/2025	Week 12	20yo presenting with palpitations; https://fo	https://drive.google.com/file/d/1GvD_anKupfgcciJP51kXxdt2H10mRZoU/view?usp=sharing
	4/3/2025	Week 13	1yo presenting for vomiting/diarrhea; https	https://drive.google.com/file/d/1HFhsj1mO1OG1_ujiPBCi4ODNYFoao-o2/view?usp=sharing
	4/10/2025	Week 14	60yo presenting for 15hours of CP; https:/	https://drive.google.com/file/d/160WzVdu2z3WubyTP7ObupSvIuA2SerxW/view?usp=sharing
	4/17/2025	Week 15	45yo presenting with shock, no history; htt	https://drive.google.com/file/d/1olqdZZ-3QW613wSqt5NzpjvOGu_jtw-D/view?usp=sharing
	4/25/2025	Week 16	45yo presenting with CP ~2hours ago that	https://drive.google.com/file/d/1x6wgcdxqEfZPJpQ4jLeLNbGCx6HQXedT/view?usp=sharing
	5/1/2025	Week 17	55yo presenting with 30min of CP; https://	https://drive.google.com/file/d/1Z9ZIsPSaOtNO20pxsxth5sJ3XNlCd57a/view?usp=sharing
	5/8/2025	Week 18	60yo presenting for 45min of CP; https://fo	https://drive.google.com/file/d/1N0CcHio5ubuspYf0I2Id6Sji9pPEr2Wj/view?usp=sharing
	5/17/2025	Week 19	45yo presenting s/p ROSC; https://forms.g	https://drive.google.com/file/d/1JRHj_prqe1JJCIMDTq6GozVpU6ARz8X7/view?usp=sharing
	5/24/2025	Week 20	35yo presenting with 20min of CP (they w	https://drive.google.com/file/d/1SCzK4vr0qgQuZxb527gAo9r8tcQqUIpD/view?usp=sharing
	5/31/2025	Week 21	80yo presenting with exertional SOB; http:	https://drive.google.com/file/d/1gwU_UAxrhzXJDHu0st8AOwzL0r42Ytiw/view?usp=sharing
	1/11/2024	Week 22	60yo presenting with shock after reporting	https://drive.google.com/file/d/1xjnFdqM2CsKsUYL1aAyvXOr7lclrVoNu/view?usp=sharing
	1/18/2024	Week 23	40yo presenting with SOB; https://forms.g	https://drive.google.com/file/d/1jcMNbz5AAHz4k0YnnoVedxJRoMI96SCO/view?usp=sharing
	1/25/2024	Week 24	70yo presenting with SOB; https://forms.g	https://drive.google.com/file/d/1gOuNZgXWbxqTt3wqCZnrZnB5PYaQj8F7/view?usp=sharing
	2/1/2024	Week 25	35yo presenting with sepsis; https://forms.	https://drive.google.com/file/d/1OI9gBCnDq1K75F0X6ZDwiUVgMbzuhUSY/view?usp=sharing
	2/8/2024	Week 26	50 presenting with CP; https://forms.gle/B	https://drive.google.com/file/d/1Kr2QBlWqqVjep4d1R4LSN_yN_Atc0tDV/view?usp=sharing
	2/15/2024	Week 27	60 presenting with CP and near syncope;	https://drive.google.com/file/d/1tQZhjPpcJN3NvppFSfllx2UZouWjySBn/view?usp=sharing
	2/22/2024	Week 28	55yo presenting with syncope; https://form	https://drive.google.com/file/d/1rWSK7HNTAYN9gaVZGgRx4TryLfyvlFq6/view?usp=sharing
	2/29/2024	Week 29	35yo presenting with palpitations; https://fo	https://drive.google.com/file/d/10-EdssarovtZmNvqD1BUch7_50r9IkWC/view?usp=sharing
	3/7/2024	Week 30	40yo presenting with LVO stroke; https://fo	https://drive.google.com/file/d/1bvJrYVHGyquEtY6IHMlLQ9F4tbGGOTUn/view?usp=sharing
	3/14/2024	Week 31	35yo presenting with palpitations, do you	https://drive.google.com/file/d/1XVI79aGybf6ZN8y6OUz-m5j07I95NOYP/view?usp=sharing
	3/21/2024	Week 32	55yo presenting with CP; https://forms.gle	https://drive.google.com/file/d/1dRAiQmR05PbUXbLVncW3_KC-cfgHHBGg/view?usp=sharing
	3/28/2024	Week 33	60yo presenting with SOB; https://forms.g	https://drive.google.com/file/d/1uu54vnvxHQP1FGIEdJ7uFV5MEWXnOntZ/view?usp=sharing
	4/4/2024	Week 34	60 presenting with SOB after CP 1 week a	https://drive.google.com/file/d/1oUYcregOlDklGmZ7X6twazwevIb_aSO2/view?usp=sharing
	4/11/2024	Week 35	55yo presenting with CP; https://forms.gle	https://drive.google.com/file/d/10rWiMWxLi4P7z4IPCQr0pLhkjEmYZmYY/view?usp=sharing
	4/18/2024	Week 36	35yo presenting with CP; https://forms.gle	https://drive.google.com/file/d/1Y09aS1TydxktkrDOpFJw0IP7bLgN4Van/view?usp=sharing
	4/25/2024	Week 37	30yo presenting for fatigue and muscle sp	https://drive.google.com/file/d/1RNNTri2ekUd5wF9h-1GTO3fz4FQNudbl/view?usp=sharing
	5/2/2024	Week 38	70yo presenting with fatigue and confusio	https://drive.google.com/file/d/1aH11_DW07Vat0wSTFRD-uBmfx83UlZ94/view?usp=sharing
	5/9/2024	Week 39	40yo presenting with...crap just look at the	https://drive.google.com/file/d/1HgvoQLe69HrUBQ63eEpURhBqbi3XRcCH/view?usp=sharing
	5/16/2024	Week 40	35yo presenting dehydrated and confused	https://drive.google.com/file/d/1qqzbDpzqP0n5RxhFdNNtqzI8hBYkwmZE/view?usp=sharing
	5/23/2024	Week 41	55yo presenting as found down outside, A	https://drive.google.com/file/d/1cmFvBSIiVxLDtEdJIWE0ZBI0e2IEWBn0/view?usp=sharing
	5/23/2024	Week 42	70yo presenting for vomiting and weaknes	https://drive.google.com/file/d/194BTMBsAt08kBmBAW5FTkB1UdcPCaPgv/view?usp=sharing
	5/30/2024	Week 43	18yo presenting with vomiting; https://form	https://drive.google.com/file/d/1yW5jPACn8Z7h-dkwZYLkdZWJhwA5_Xkd/view?usp=sharing
	6/6/2024	Week 44	18yo presenting with syncope; https://form	https://drive.google.com/file/d/15a4GBbfmrRYupx_iU3_rMPIXF77BbDDX/view?usp=sharing
	6/13/2024	Week 45	30yo presenting after VT arrest s/p ROSC	https://drive.google.com/file/d/1ByxLwIr2Jy2KwNur4MTY-JmrYG5CeNsi/view?usp=sharing
	6/20/2024	Week 46	35yo Italian presenting after syncope; http	https://drive.google.com/file/d/1jzaiolCfjdCdxNlizPXIsLdr5FC_JYN_/view?usp=sharing
	6/27/2024	Week 47	30yo presenting in shock; https://forms.gle	https://drive.google.com/file/d/1R4CTgJAV0sV511Fi8NSYAsH2CKZ41emO/view?usp=sharing
	7/4/2024	Week 48	35yo presenting with dizziness; https://form	https://drive.google.com/file/d/1uAdLeMxjRx5iTMggY41JHycpc0Wwu4T4/view?usp=sharing
	7/11/2024	Week 49	60yo presenting with CHF exacerbation; h	https://drive.google.com/file/d/1JFifjBqVmYCwv5r_Rl4jDsGdx1XMduol/view?usp=sharing
	7/18/2024	Week 50	60yo presenting with shortness of breath;	https://drive.google.com/file/d/1VgofbJ-GU2-BAjmrRC7UkBoGzo1WtFOt/view?usp=sharing
	7/25/2024	Week 51	60yo presenting with shortness of breath;	https://drive.google.com/file/d/11Cs-awbFpwtgCnHV5vtf0CVqNU7MAIK4/view?usp=sharing
	8/1/2024	Week 52	60 presenting with palpitations, CP, and S	https://drive.google.com/file/d/18uleL34x4HQB4n48V663dJ7ERFf_1qBs/view?usp=sharing
	8/8/2024	Week 53	50yo presenting with 'my afib'; https://form	https://drive.google.com/file/d/1m_Fiky4c7t33_uqh-q9af-sKS8QI7rsB/view?usp=sharing
	8/15/2024	Week 54	70yo hx CAD presenting after syncope, nu	https://drive.google.com/file/d/1pbAwSkt6LIde29_qlNdG4ygnrDWMC6gm/view?usp=sharing
	8/22/2024	Week 55	55yo presenting with HA, AMS, and bilater	https://drive.google.com/file/d/1hMya2yYMdynQrMdJ7Drr8WgtSNiFAJor/view?usp=sharing
	8/29/2024	Week 56	40yo presenting with respiratory distress;	https://drive.google.com/file/d/1eYWEb0jKEVZP9eBxvgH9kMopOsmeyhbM/view?usp=sharing
	9/5/2024	Week 57	30yo presenting with vomiting and diarrhe	https://drive.google.com/file/d/12p5arEgaLWj2UA1c3EdAmHjsalAjKnZZ/view?usp=sharing
	9/12/2024	Week 58	35yo presenting with syncope; https://form	https://drive.google.com/file/d/12M3LrC2N5felaKalz7puQO0J7flEAvt0/view?usp=sharing
	9/19/2024	Week 59	25yo presenting with CP; https://forms.gle	https://drive.google.com/file/d/17A4UbXNcbcyVp5ENF4o758y_O30xYKSz/view?usp=sharing
	9/26/2024	Week 60	45yo presenting with SOB and LE edema;	https://drive.google.com/file/d/1kUu94MYslQLqbmEEg-4f3PbfrZkceaHW/view?usp=sharing
	10/3/2024	Week 61	45yo presenting with CP; https://forms.gle	https://drive.google.com/file/d/1GQIVPODRh-SnvGQw4KL6JLeZwLXF6HT1/view?usp=sharing
	10/10/2024	Week 62	35yo presenting with CP; https://forms.gle	https://drive.google.com/file/d/1MHq52FZKKX92jRv2ANQE8_32GiE-pg2D/view?usp=sharing
	10/17/2024	Week 63	60yo presenting with SOB; https://forms.g	https://drive.google.com/file/d/1BUs-3EPXEPTnHuqwhz-anAM15m60Ld57/view?usp=sharing
	10/24/2024	Week 64	60yo presenting with syncope; https://form	https://drive.google.com/file/d/1-IAB_1n5T-LipTD_4ZDbWKd6sOKQbwJq/view?usp=sharing
	10/31/2024	Week 65	60yo presenting with nausea and vomiting	https://drive.google.com/file/d/1I2m5q7UxwmR3z9jXsPY2I9IM7K5rwMdo/view?usp=sharing
	11/7/2024	Week 66	90yo presenting with tachycardia; https://fo	https://drive.google.com/file/d/1Ud_VK6MFx3gKJ-H7xjjpcvdJeKuAoJkJ/view?usp=sharing
	11/14/2024	Week 67	Nursing asks you to see a patient in this ro	https://drive.google.com/file/d/1QgZQ6TyClQVyoHZdBnJCAO-xc3cCxsy3/view?usp=sharing
	11/21/2024	Week 68	35yo psych patient with vomiting; https://fo	https://drive.google.com/file/d/1X1dqUSgVKjLkItcdNtsMkwyR_Uylx9ka/view?usp=sharing
	11/28/2024	Week 69	40yo presenting with palpitations; https://fo	https://drive.google.com/file/d/1NsXSVF7enYVayvVjE2PXNIruoAEPFRzo/view?usp=sharing
	12/5/2024	Week 70	40yo presenting with palpitations; https://fo	https://drive.google.com/file/d/1KsTWbfj0fXvgYkF_-uh4GNCiiOtIj0fr/view?usp=sharing
	12/12/2024	Week 71	35yo presenting with palpitations; https://fo	https://drive.google.com/file/d/1rOs8Xny4RQX5VOAuryxzOB0I7H6fpDVJ/view?usp=sharing
	12/19/2024	Week 72	50yo presenting with SOB; https://forms.g	https://drive.google.com/file/d/1fo8XBqRqvbY2aP2oCXztDtVn7a4R2CTU/view?usp=sharing
	12/26/2024	Week 73	45yo presenting with chest pain and palpit	https://drive.google.com/file/d/1zwLW2GwlH6J9EtxL5rZJXNgYAYMyzgYj/view?usp=sharing
	1/2/2025	Week 74	18yo presenting for palpitations; https://for	https://drive.google.com/file/d/1IzcRXUGdkLMrJm4zkNC_U0yecXqnxkCZ/view?usp=sharing
