# Supplementary material for: Enhancing Emergency Medicine Resident Education: A Weekly Education Series to Augment Electrocardiogram Education
Source: J Educ Teach Emerg Med. 2026 Jan 31;11(1):C1–C89. doi: 10.5070/M5.52141 (PMC12880887; doi:10.5070/M5.52141)
Supplement: Supplementary file 2 [file 11-1-C1-AppendixC.pdf]

I

NORMAL PACEMAKER

Pacemakers are not the same as AICD. They are classified by how they pace, with 5 different letters potentially (though only the first 3 are commonly used). The most common are VVI and DDD. The second image shows a pathway to determine the type of pacemaker if the patient does not have their card.

Position I	Position II	Position III	Position IV	Position V
Chamber(s)Paced	Chamber(s)Sensed	Response to Sensing	Rate Modulation	Multisite Pacing
O = None	O = None	O = None	O = None	O = None
A = Atrium	A = Atrium	T = Triggered	R = Rate Modulation	A = Atrium
V = ventricle	V = ventricle	I = Inhibited		V = ventricle
D = Dual (A+V)	D = Dual (A+V)	D = Dual (T+I)		D = Dual (A+V)

Source: LITFL

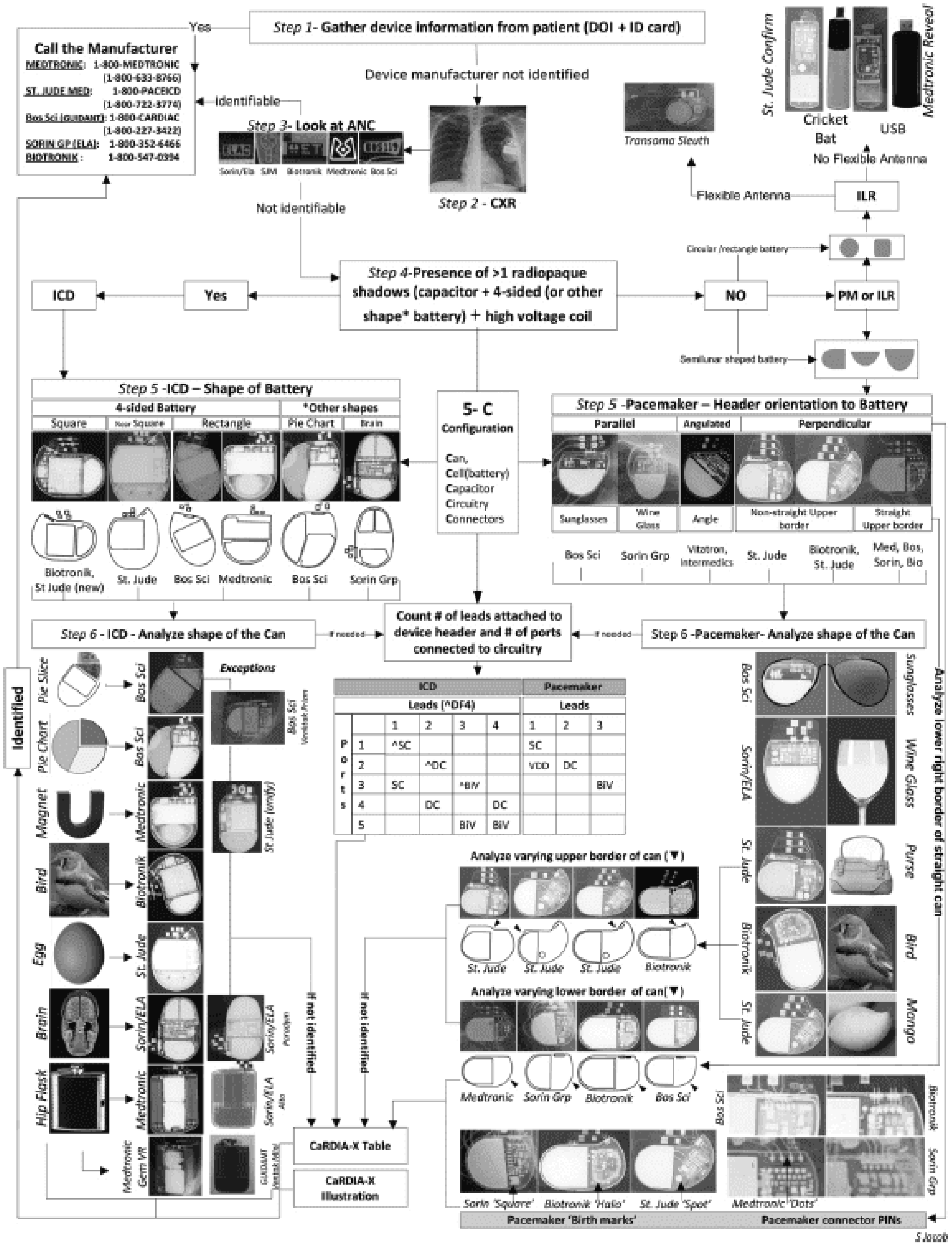

Source: Rebel EM

The leads are typically placed on the right side (right atrium and right ventricle). VVI is a right ventricular lead that senses for native ventricular electrical activity and, when this is not present sufficiently to inhibit the pacemaker, the ventricular lead will initiate a depolarization. DDD, then, is where the atrial lead and ventricular lead are both sensing for atrial and ventricular depolarizations, respectively; they will also pace their respective chambers when the underlying conduction is not sufficient. If the underlying/native electrical activity is sufficient, the pacemaker should not be firing (no pacemaker spikes in a patient with sufficient electrical activity is still normal for patients with pacemakers). The ventricular component frequently waits 200ms before initiating a spike. Applying a magnet to a pacemaker initiates 'magnet mode'; these effects can vary by specific pacemakers, though typically this produces an asynchronous pacing mode, where the pacemaker consistently delivers depolarizations at a set rate regardless of the native electrical activity. In other words, the native electrical activity may be sufficient, but the pacemaker ignores this and still provides pacing. The asynchronous modes are usually AOO, VOO, or DOO. It is important to note that these devices are typically programmed to have certain pacing and sensing thresholds that are not always effective for preventing pathology. For example, patients can have VT that is not 'taken care of' by the pacemaker if it is slow enough and the sensing threshold of the pacemaker is higher than this.

Pacing is shown on an EKG with pacer spikes, which are vertical lines on the EKG. For atrial pacing, these vertical lines (usually smaller) should be just before a P wave. For ventricular pacing, these vertical lines (usually larger) should be just before a QRS. These spikes are often not easily seen in all leads. New pacemakers tend to produce smaller pacemaker spikes as well. The ventricular spikes produce a wide QRS complex. One can delineate which chamber the ventricular lead is by identification of the bundle branch block that is produced. For example, a lead placed in the right ventricle (most common) produces a left bundle branch block appearance of the QRS (depolarization starts on right and moves to left). However, these bundle branch block patterns are not always completely classic; for example, the LBBB pattern has typical QS pattern in right-sided chest leads, but still has negative QRS complexes in the lateral leads. Because these ventricular pacemaker spikes produce a depolarization abnormality, there will also be repolarization abnormalities that follow expected discordance patterns. Because of this, Sgarbossa criteria can be used for identification of MI in the setting of a V-paced rhythm. Atrial-paced rhythms not requiring ventricular pacing will produce a QRS complex that is the patient's normal QRS complex and, therefore, will not have repolarization abnormalities unless the patient normally has a bundle branch block.

One last discussion of pacemaker basics involves cardiac resynchronization therapy. CRT is also known as biventricular pacing. Obviously, there is a lead in each ventricle with this to synchronize the ventricular depolarizations (hence resynchronization). Patients typically have this done when they have a wide LBBB with poor cardiac output as resynchronizing the ventricles helps to improve left ventricular output by having LV preload actually enter the LV during diastole as opposed to only partially in diastole and partially in systole. Because there is a lead in each ventricle, there is not a specific bundle branch block pattern, though there is a wide QRS with appropriate discordant ST-T changes.

Examples:

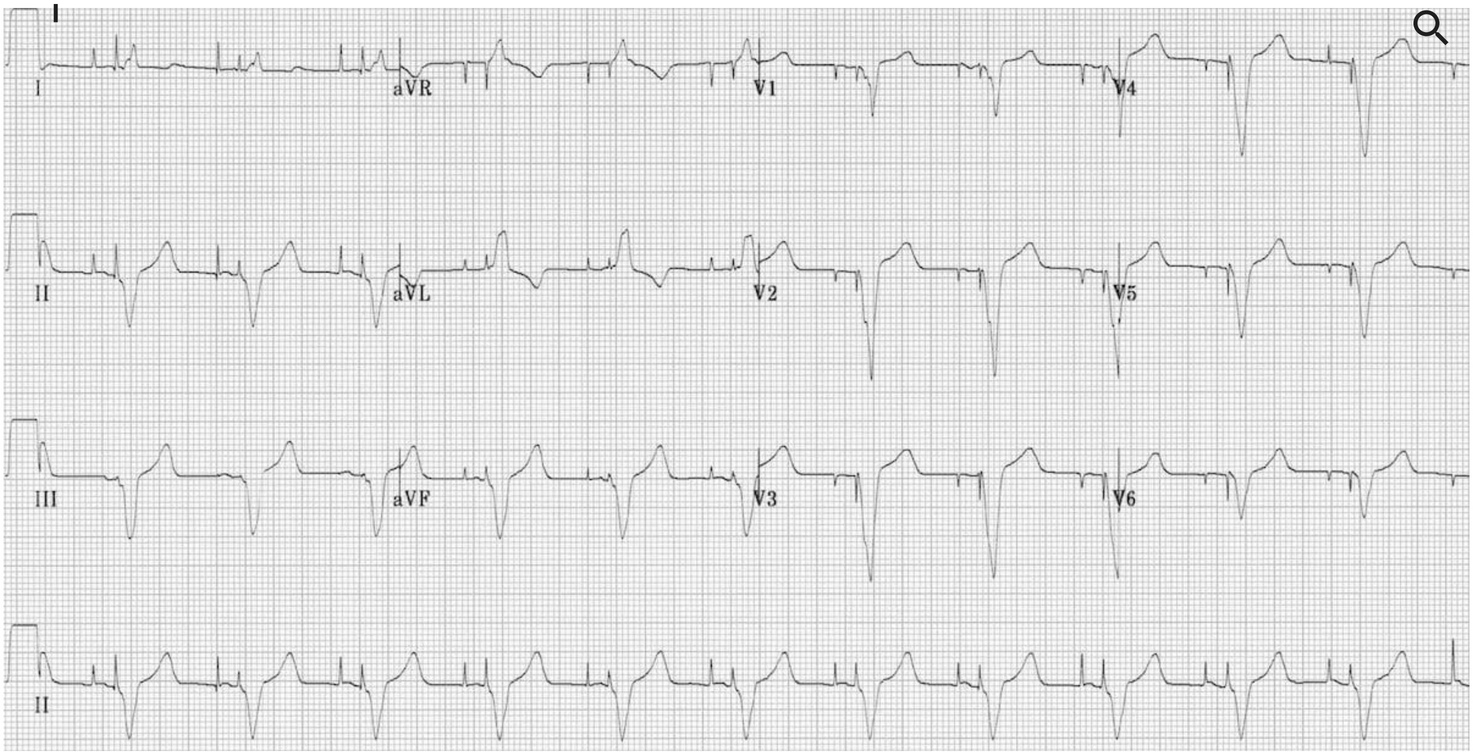

Source: LITFL

An A-V sequentially paced rhythm. Note both atrial and ventricular spikes with 100% capture. After the atrial spike, there are subsequent P waves (difficult to see, though evidence in inferior leads). After the ventricular spike, a wide QRS complex is formed closer to the LBBB pattern (this is typical 'LBBB pattern' with ventricular pacing).

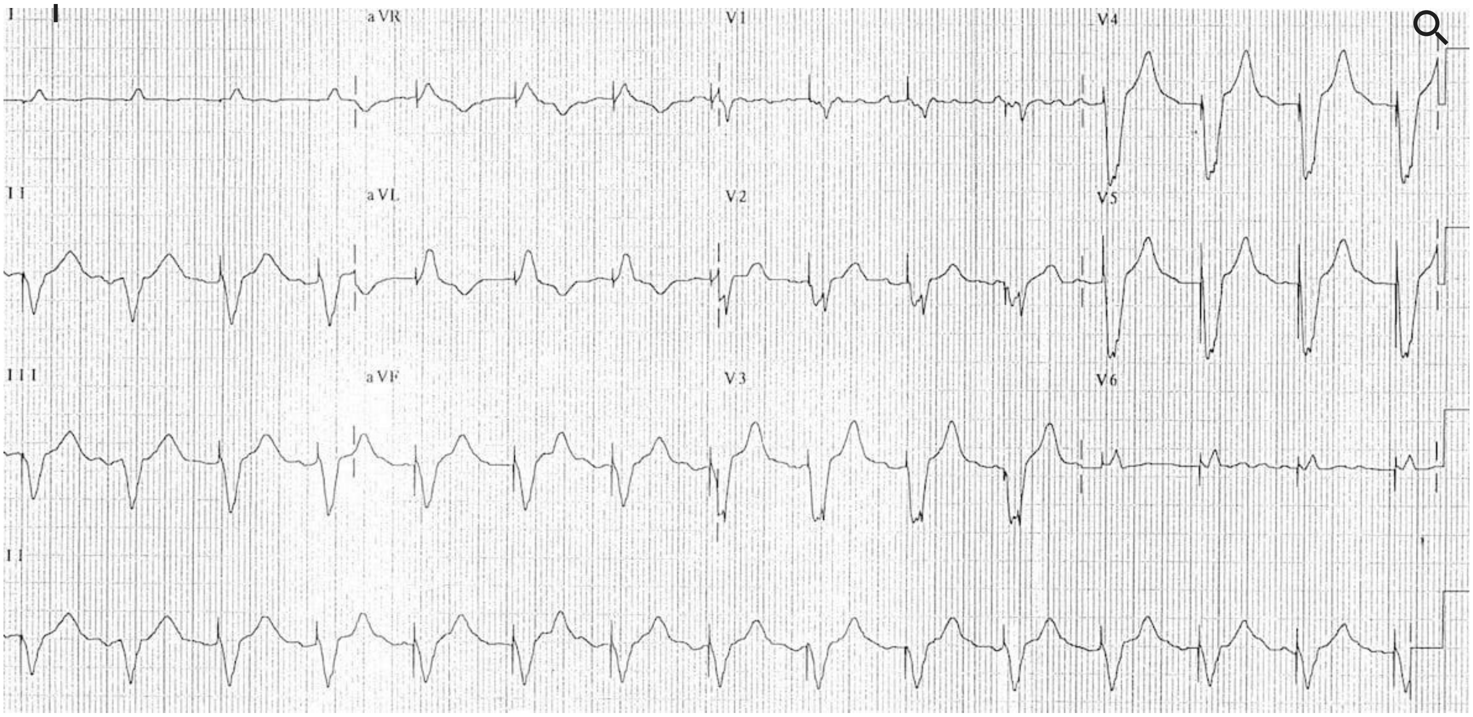

Source: LITFL

A V-paced rhythm with ventricular spikes preceding wide QRS complexes typical of a LBBB-type pattern. Note the typical repolarization abnormalities (expected discordance) that are not evidence of ischemia. This patient likely has a VVI pacemaker. If this were a DDD with atrial sensing but lack of atrial spikes, there should be necessary native atrial activity (ie necessary P waves) that are simply not present. The underlying atrial activity is like atrial fibrillation given the appearance of V1 with what appears to be course P waves. If this were DDD with atrial fibrillation without atrial spikes, then the ventricular pacing should be irregularly irregular as the atrial impulses will be irregularly irregular.

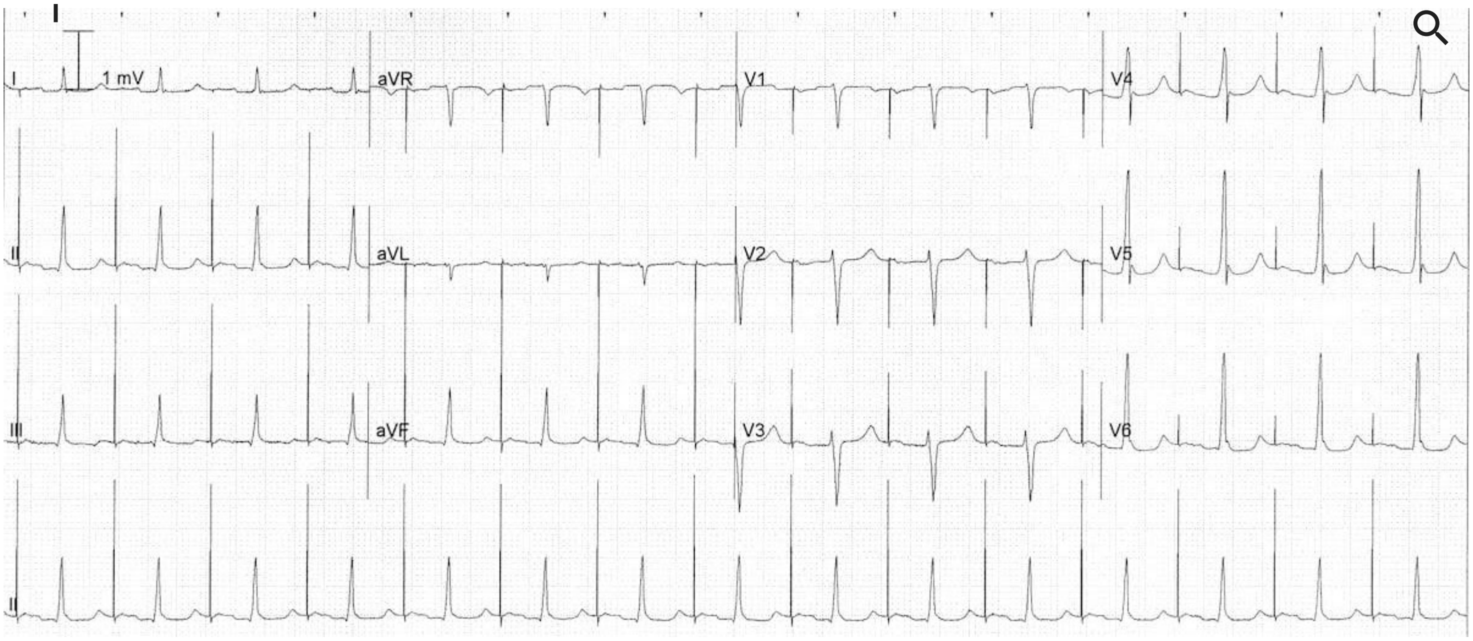

Source: LITFL

Atrial-paced rhythm with 100% atrial capture (P waves follow every atrial spike). There are no ventricular spikes. Although we technically cannot say that there is a ventricular lead without ventricular spikes, this is a DDD given the pacemakers that are placed. There are no ventricular spikes as the underlying ventricular conduction sensed by the ventricular lead is adequate to inhibit ventricular pacing.

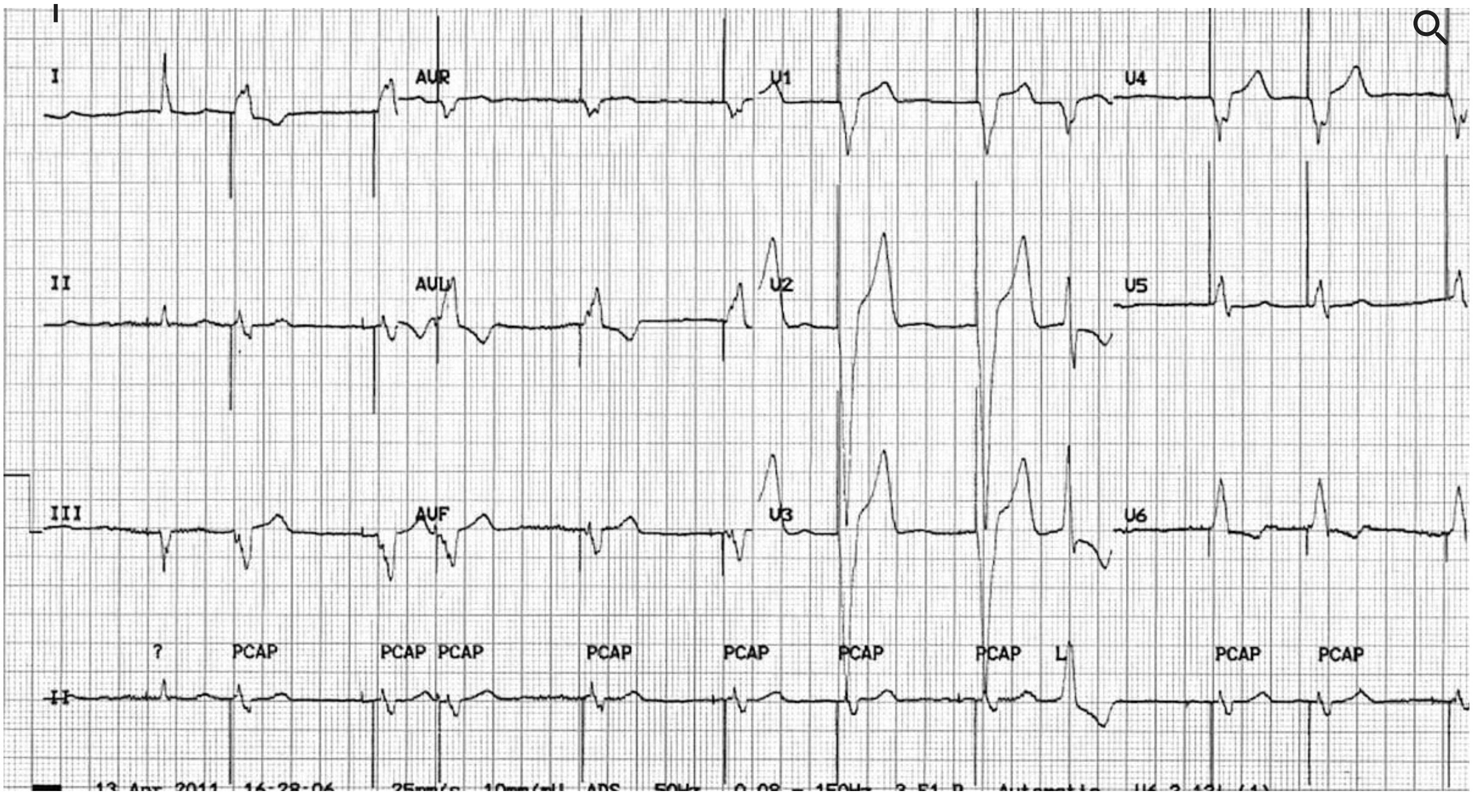

Source: LITFL

Variable atrial and ventricular spikes (DDD pacemaker). Looking at the rhythm strip, only 2 of the QRS complexes are not preceded by ventricular spikes. The first beat has an atrial spike with a narrow complex QRS as the native ventricular electrical activity produces the narrow complex QRS and, therefore, inhibits the ventricular pacing. Beat 9 also does not have ventricular pacing though is wide; it is a PVC. About half of the other beats have both atrial and ventricular pacing. Beats 2, 4, 5, 7, and 11 have ventricular spikes without atrial spikes; this is because the atrial lead senses underlying atrial activity so does not fire, but the underlying ventricular activity in these beats is not adequate so the ventricular lead fires. Because no clear atrial activity is seen in these beats, the underlying activity is likely atrial fibrillation. This EKG does not demonstrate any pacemaker pathology.

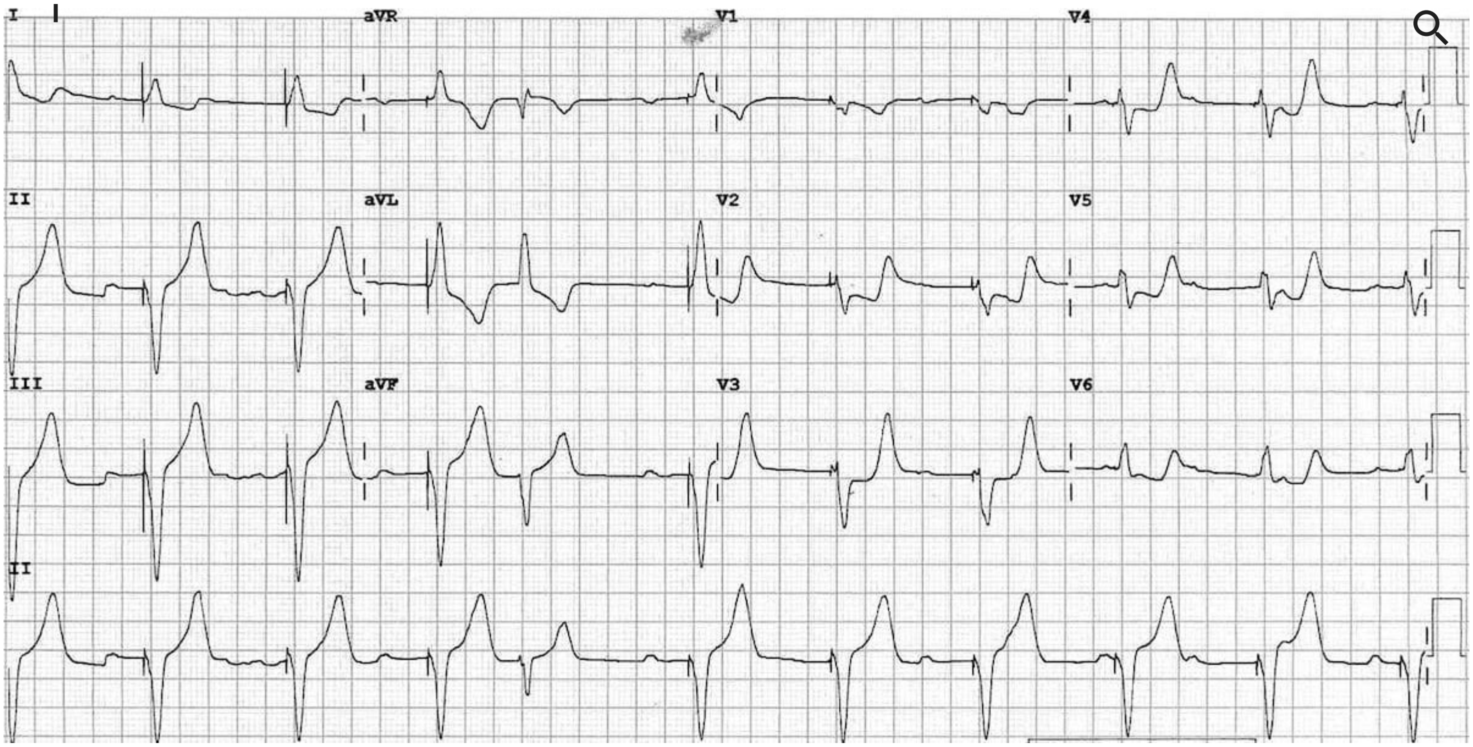

Source: LITFL

V-paced rhythm with concordant ST depressions in V2-V5 consistent with STEMI. Beat 5 does not have notable pacemaker spikes as the underlying native rhythm is sufficient to inhibit the pacemaker.

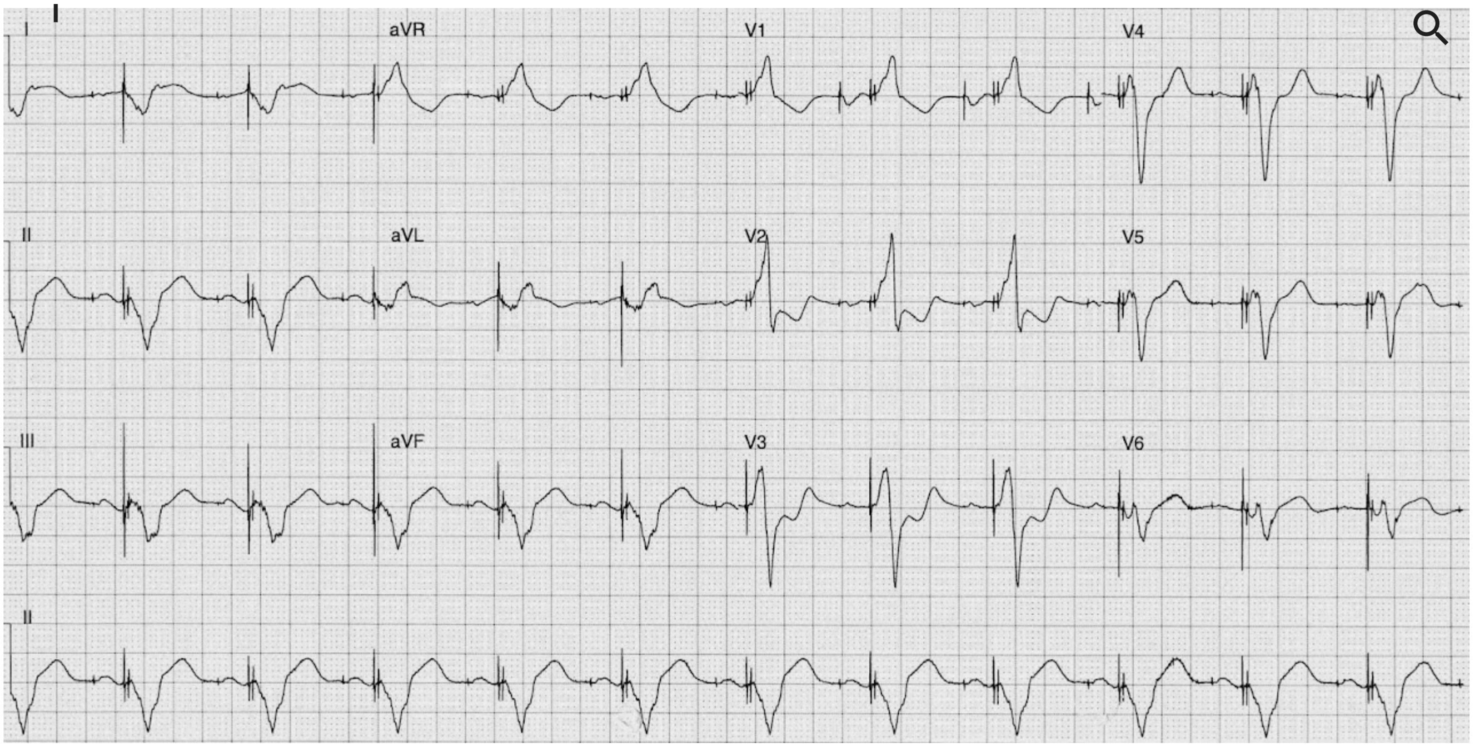

Source: ECG of the Week

A-V sequentially paced rhythm. There are small atrial spikes followed P waves. There are also 2 ventricular spikes (common to have slight delay between the 2 ventricular spikes) followed by a wide QRS without a specific bundle branch block pattern.

Further Reading:

https://litfl.com/pacemaker-rhythms-normal-patterns/

http://hqmeded-ecg.blogspot.com/search/label/paced%20rhythm

http://hqmeded-ecg.blogspot.com/search/label/Appropriate%20Discordance%20in%20LBBB%20and%20Paced%20Rhythm

https://rebelem.com/pacemaker-basics/

This website does not provide medical advice.

This site is not an official website for any hospital system or enterprise but is an educational website meant to assist in the education of emergency medicine residents, medical students, and faculty.

The content of this website, such as graphics, images, text and all other materials, is provided for reference and educational purposes only. The content is not meant to be complete or exhaustive or to be applicable to any specific individual's medical condition.
